# Genetic characterization of preschool wheeze phenotypes

**DOI:** 10.1016/j.jaci.2025.07.015

**Published:** 2025-08-05

**Authors:** Kasper Fischer-Rasmussen, Raquel Granell, Anders Ulrik Eliasen, Eskil Kreiner, Casper-Emil Tingskov Pedersen, Yang Luo, Bo Chawes, Jakob Stokholm, Ann-Marie Malby Schoos, Ashish Kumar, Anne-Marie Nybo Andersen, Bjarke Feenstra, Frank Geller, Valérie Siroux, Florence Demenais, Emmanuelle Bouzigon, Vincent Jaddoe, Ralf J. P. van der Valk, Liesbeth Duijts, Jordi Sunyer, Mónica Guxens, Marcella Marinelli, Mariona Bustamante, Joachim Heinrich, Marie Standl, John Curtin, Angela Simpson, Clare Murray, Bo Jacobsson, Ronny Myhre, Craig E. Pennell, Denise Daley, Carole Ober, James E. Gern, Daniel Jackson, Dorret I. Boomsma, Jouke-Jan Hottenga, Abdel Abdellaoui, John W. Holloway, Sam Collins, Stephen Turner, S. Hasan Arshad, Anhar Ullah, Erik Melén, John Henderson, Hans Bisgaard, Anders Gorm Pedersen, Adnan Custovic, Judith M. Vonk, Gerard H. Koppelman, Michael Kabesch, Klaus Bønnelykke

**Affiliations:** a Copenhagen Prospective Studies on Asthma in Childhood (COPSAC), Herlev and Gentofte Hospital, University of Copenhagen, Copenhagen; b the Department of Health Technology, Section for Bioinformatics, Technical University of Denmark, Kongens Lyngby; c the MRC Integrative Epidemiology Unit, Population Health Sciences, Bristol Medical School, University of Bristol, Bristol; d the Department of Clinical Medicine, Faculty of Health and Medical Sciences, University of Copenhagen, Copenhagen; e the Department of Pediatrics, Copenhagen University Hospital - Amager and Hvidovre, Hvidovre; f the Department of Pediatrics, Copenhagen University Hospital - Næstved, Slagelse and Ringsted, Slagelse; g the Department of Clinical Science and Education, Södersjukhuset, Karolinska Institutet, Stockholm; h the Sach’s Children and Youth Hospital, Södersjukhuset, Stockholm; i the Department of Public Health, University of Copenhagen, Copenhagen; j the Department of Epidemiology Research, Statens Serum Institut, Copenhagen; k the Université Paris Cité, INSERM, UMR 1124, Group of Genomic Epidemiology of Multifactorial Diseases, Paris; l The Generation R Study Group; m the Department of Pediatrics, Division of Respiratory Medicine and Allergology; n the Department of Neonatal and Pediatric Intensive Care, Division of Neonatology, Erasmus MC, University Medical Center Rotterdam, Rotterdam; o ISGlobal, Barcelona; p the Spanish Consortium for Research on Epidemiology and Public Health (CIBERESP), Madrid; q the Universitat Pompeu Fabra (UPF), Barcelona; r the CIBER Epidemiología y Salud Pública, Madrid; s ICREA, Barcelona; t the Hospital del Mar Research Institute (IMIM), Barcelona; u the Center for Research in Environmental Epidemiology (CREAL), Barcelona; v the Division of Metabolic and Nutritional Medicine, Ludwig-Maximilians-Universitat (LMU) Munich, Dr von Hauner Children’s Hospital, University of Munich Medical Center, Munich; w the Institute and Clinic for Occupational, Social and Environmental Medicine, University Hospital, LMU Munich, Munich; x the Institute of Epidemiology, Helmholtz Zentrum München, German Research Center for Environmental Health, Neuherberg; y the German Center for Lung Research (DZL); z the German Center for Child and Adolescent Health (DZKJ), Munich; aa the Division of Immunology, Immunity to Infection and Respiratory Medicine, Manchester Academic Health Science Centre, School of Biological Sciences, University of Manchester, Manchester; ab the National Institute for Health Research (NIHR) Manchester Biomedical Research Centre, Manchester University Hospital NHS Foundation Trust, Manchester; ac the Department of Genes and Environment, Norwegian Institute of Public Health, Oslo; ad the Department of Obstetrics and Gynecology, Sahlgrenska Academy, Gothenburg; ae the Hunter Medical Research Institute; af the Mothers and Babies Research Centre and John Hunter Hospital, New Lambton Heights; ag the School of Medicine and Public Health, College of Health, Medicine and Wellbeing, University of Newcastle, Callaghan; ah the Center for Heart and Lung Innovation; ai the University of British Columbia, Faculty of Medicine, Vancouver; aj the Department of Medicine, University of British Columbia, Vancouver; ak Pediatrics, School of Medicine and Public Health, University of Wisconsin, Madison; al the Department of Human Genetics, University of Chicago, Chicago; am the Department of Biological Psychology, Vrije Universiteit Amsterdam, Amsterdam; an the NIHR Southampton Biomedical Research Centre, University of Southampton and University Hospital Southampton NHS Foundation Trust, Southampton; ao Human Development and Health; ap Clinical and Experimental Sciences, University of Southampton Faculty of Medicine, Southampton; aq Child Health, University of Aberdeen, Aberdeen; ar the Women and Children Division, NHS Grampian, Aberdeen; as the National Heart and Lung Institute; at Pediatric Allergy, Imperial College London, London; au the Groningen Research Institute for Asthma and COPD (GRIAC); av the Department of Pediatric Pulmonology and Pediatric Allergology, Beatrix Children’s Hospital, University of Groningen, University Medical Center Groningen, Groningen; aw the Department of Pediatric Pneumology and Allergy, St Hedwig’s Hospital of the Order of St John, University Children’s Hospital Regensburg (KUNO), Regensburg.

**Keywords:** Preschool wheeze, 17q21–12, genome-wide association study, genetic overlap, asthma comorbidities

## Abstract

**Background:**

Preschool wheeze is a heterogenous and poorly understood clinical syndrome. As a result, current treatments are insufficient, and prevention is not possible.

**Objective:**

We sought to increase understanding of the genetic susceptibility and underlying disease mechanisms of wheeze phenotypes in early childhood through large-scale genome-wide association study analyses.

**Methods:**

We performed meta-analyses of genome-wide association study on early-onset wheeze, defined as recurrent wheeze or asthma in the first 3 years of life, and its subtypes, including early transient and persistent wheeze, defined by asthma/wheeze at age 3 and subsequent remission or persistence at age 6, respectively. The discovery analyses included data on more than 13,000 children from 15 cohorts; replication was sought through meta-analyses of data from 7 additional cohorts including up to 5000 children. Genetic variants associated with asthma-related traits in adulthood (adult asthma, atopy, eosinophils, and lung function) were used to quantify the degree to which genetic risk influencing asthma-related adult traits also influences genetic risk of preschool wheeze.

**Results:**

Variants near the *GSDMB* gene in the 17q region showed genome-wide significant association with early-onset wheeze (rs2305480; odds ratio [95% confidence interval] = 1.26 [1.17–1.33], *P* = 2.30E-16) and persistent wheeze (rs11078926; 1.43 [1.30–1.578], *P* = 2.14E-11), but not with early transient wheeze (rs1054609; 1.08 [0.98–1.18], *P* = .094). Other known asthma loci were associated with early-onset wheeze, particularly *CDHR3*. Additionally, increased genetic risk to early-onset wheeze was associated with genetic risk for asthma at older ages, atopy, eosinophil count, and lower adult lung function. This was driven by persistent wheeze, whereas transient early wheeze was only associated with low lung function.

**Conclusions:**

Preschool wheeze phenotypes displayed distinct patterns of single nucleotide polymorphism associations and genetic enrichment with asthma-related traits. These results indicate distinct etiologies of wheeze phenotypes, which could inform studies in optimization of prevention and treatment strategies. (J Allergy Clin Immunol 2025;156:1537–46.)

Asthma and asthma-like symptoms occurring in children of preschool age, often termed *preschool wheeze,* is a heterogenous and poorly understood clinical syndrome with both genetic and environmental etiologies. It is common in childhood as the most frequent cause of acute hospitalizations in early life, and it is associated with a substantial burden on quality of life for affected children and families as well as socioeconomic costs for society.^1–4^ The incomplete understanding of recurrent preschool wheeze is probably a main reason for current treatment insufficiency and prevention of the disorder.^3^ *Wheeze* is often used to describe symptoms such as whistling in the chest while breathing and can arise from various conditions such as bronchitis or bronchiolitis, triggered by lower respiratory tract infections, especially rhinovirus.^5^ Furthermore, recurrent wheeze in preschool children is a strong risk factor for asthma at school age.^6^

Evidence from twin studies estimates heritability to be as high as 80% for childhood asthma and 60% for ever having wheezed during childhood.^7,8^ Multiple susceptibility loci have been discovered in genome-wide association studies (GWAS) on asthma,^9–14^ but little is known regarding genetic determinants of preschool wheeze.^15^ Using specific phenotype definitions may have improved power for discovery of susceptibility variants and thereby may elucidate underlying mechanisms.^15^ This is exemplified by previous studies of severe exacerbations in preschool children identifying *CDHR3* (cadherin-related family member 3) as a novel susceptibility gene,^9^ and of longitudinal wheeze phenotypes from birth to 18 years identifying *ANXA1* as associated with persistent wheeze,^16^ both of which were not discovered in larger GWAS using less specific definitions of asthma.

In this study, we performed a GWAS using data from the EAGLE (EArly Genetics and Lifecourse Epidemiology) and GABRIEL (Multidisciplinary Study to Identify the Genetic and Environmental Causes of Asthma) consortia^17,18^ in order to create what is to our knowledge the largest study of temporal wheeze phenotypes in early life, comprising data from more than 18,000 children from 22 studies. We examined early-onset wheeze, defined as recurrent wheeze or asthma in the first 3 years of life, as well as subtypes of early-onset wheeze in terms of transient early wheeze, with symptoms resolving before age 6, and persistent early wheeze, with symptoms persisting to age 6. We also investigated current asthma by age 6, defined as cross-sectional asthma at age 6 irrespective of age at onset. Because of the small number of children with late-onset wheeze across the studies, a specific GWAS on this phenotype was not performed. Our objective was to characterize genetic differences and similarities among the 4 phenotypes of preschool wheeze to illuminate underlying molecular mechanisms.

## METHODS

### Preschool wheeze phenotypes

Preschool wheeze was defined as 3 or more wheezing episodes or an asthma diagnosis before the age of 3. Transient early wheeze (TEW) was defined as preschool children with wheeze who were free of symptoms between ages 5 and 7 years. Persistent early wheeze (PEW) included children with preschool wheeze and one wheezing episode or an asthma diagnosis between ages 5 and 7 years. Early-onset wheeze (EOW) consisted of both TEW and PEW. Current asthma (CA) was defined by at least one wheezing episode or asthma diagnosis between ages 5 and 7 years, irrespective of age at onset, thus including both PEW and children with late onset of wheeze or asthma (after 3 years) (see Table E1 in the Online Repository available at www.jacionline.org). All children not fulfilling the case criteria were used as controls for any subsequent phenotype. This approach was chosen to ensure phenotype-specific signals by avoiding potential bias otherwise introduced by excluding children with other asthma/wheeze phenotypes or related traits from the control group. Late-onset wheeze or asthma was not analyzed separately in this study because there were too few cases.

### Discovery analysis

A total of more than 13,000 individuals of European descent were recruited from 15 separate research initiatives, all participating in the GABRIEL and EAGLE consortia.^17,18^ Ethical approval was granted by local research ethics committees for each individual study, and participants or child’s legal guardians provided informed consent, where applicable. Genotyping and subsequent imputation was conducted within each study. Association analysis was executed by logistic regression models, implementing an additive model for single nucleotide polymorphisms (SNPs). Variants were excluded in the meta-analysis if they occurred in fewer than 3 studies, had a minor allele frequency below 5%, or did not occur in the 1000 Genomes Project reference panel.^18^ Table E2 in the Online Repository available at www.jacionline.org provides inclusion criteria, quality control, and imputation of discovery cohorts. Fixed-effects inverse-variance weighted meta-analysis was performed by METAL software (version released May 5, 2020),^19^ producing heterogeneity statistics to account for cohort-level differences such as genotyping platform and imputation tools. Functionally informed *Z*-score imputation was used to impute summary results to increase genotype density. *Z* scores were converted to log odds ratios (logOR) and standard errors using equations from Zhu et al^20^ (see the supplementary material in the Online Repository available at www.jacionline.org).

### Replication analysis

Variants were carried forward to the replication phase on the basis of two criteria. First, the *P* value should be less than =e-5. Second, the variant must be in a locus not previously known to be associated with asthma. Because of the attenuated signal at the standard genome-wide significance cutoff (5e-8), we utilized a more liberal *P*-value cutoff (5e-5) in the discovery analysis, allowing about 5% false-positive results among the selected variants.^21^ Replication was sought in more than =000 individuals of European descent recruited from 7 different studies. Informed consent was gathered from the parents in each individual replication study. The replication studies carried out their own genotyping and quality control. Table E3 in the Online Repository available at www.jacionline.org provides inclusion criteria, quality control, and imputation of replication cohorts.

### Analysis of known asthma variants

To investigate whether known asthma variants were associated with the wheeze phenotypes, we gathered lead variants from previous GWASs on asthma, including phenotypes of asthma ever,^11,22,23^ adult asthma, childhood asthma,^13,14^ and early childhood asthma with severe exacerbations.^9,12^ This resulted in 207 independent variants (*r*^2^ < 0.01) available for association with EOW, TEW, PEW, and CA. Variants showing similar direction of effect and a false discovery rate (FDR) below 5% were considered to be associated with preschool wheeze. Correction for multiple testing was performed by the Benjamini-Hochberg method.

### Genetic similarity between adult traits and preschool wheeze

Obtaining reliable estimates of genetic correlation (shared heritability) using linkage disequilibrium (LD) score regression^24^ was not feasible in this study. Instead, we leveraged a method for Mendelian randomization to approximate genetic correlation because the wheeze phenotypes displayed weak heritability (see Table E4 in the Online Repository available at www.jacionline.org). Previous studies have shown that methods for Mendelian randomization are confounded by genetic correlation, and Mendelian randomization and genetic correlation answer similar questions.^25^ To characterize the genetic similarity of phenotypes with related traits, we instead utilized GSMR (Generalised Summary-data–based Mendelian Randomisation),^26^ a tool originally developed for Mendelian randomization analysis to compare association coefficients of alleles between traits from published GWAS and GWAS of preschool wheeze. GSMR was selected for its ability to accurately account for sampling variance in both exposure and outcome GWAS, which was particularly important given the limited power the wheeze GWAS. We omitted the HEIDI-outlier analysis because our study did not aim to establish causal inference (see the supplementary code provided in the Online Repository). The related traits were included based on published large-scale GWASs on childhood-onset asthma, adult-onset asthma,^14^ asthma ever,^23^ severe exacerbations in early childhood,^9,12^ allergic sensitization,^27^ atopic dermatitis,^28^ eosinophil count,^29^ and lung function measurements.^30^ Detailed descriptions of phenotype definitions of included traits are provided in the Online Repository. Related traits were included as exposures and the wheeze phenotypes as outcomes; the genetic overlap can be interpreted as a logOR for wheeze status, given a unit increase in the genetic risk of the related traits.^26^ Correction for multiple testing was performed by the Benjamini-Hochberg method.

### Lung development genes

To investigate if the wheeze phenotypes were associated with genetic risk factors involved in lung development, we aimed to characterize variants located near genes previously known to be involved in lung development. The list of genes was compiled as described in Portas et al.^31^ We mapped SNPs to genes based on proximity by BEDTools software.^32^

## RESULTS

### Discovery analyses

We included 3273 children with EOW, 977 who also had wheeze at age 6 (PEW), and 1338 whose wheeze remitted (TEW). The symptom trajectory for the remaining 2335 preschool children with wheeze was unknown across the discovery cohorts. We included 2104 children with CA (Table I). The only locus that reached genome-wide significance in the GWAS was the known 17q12–21 locus (17q). Variants at this locus were associated with 3 of the 4 phenotypes, namely EOW, PEW, and CA (Table II). We did not find any genome-wide significant loci for TEW (see Fig E1 and E2 in the Online Repository available at www.jacionline.org). The lead variant in the 17q region for PEW (rs11078926; odds ratio [OR] [95% confidence interval (CI)] 5 1.43 [1.30–1.578], *P* = 2.14E-11), EOW (rs2305480; 1.26 [1.17–1.33], *P* = 2.30E-16), and CA (rs1008723; OR 1.38 [1.30–1.52], *P* = 1.75E-17) were all strongly correlated (minimum *r*^2^ = 0.82) (see Table E5 in the Online Repository). All suggestive SNPs within a 1 Mb window (*r*^2^ < 0.01) of a suggestive locus are shown in Table E6, and the number of additional SNPs obtained from functionally informed *Z*-score imputation is shown in Table E7, both available in the Online Repository.

### Replication analysis

For replication, we included 1235 children with EOW, with 471 who also had wheeze at age 6 (PEW) and 734 whose wheeze remitted (TEW), with only 30 children with unknown wheeze trajectories. We included 725 children with CA (Table I). Ten suggestive SNPs (*P* < 5e-5) in the discovery phase were unknown asthma loci and were carried forward to the replication phase (Table II). None of these SNPs showed evidence of replication in independent cohorts. One variant, rs2214823 near *HGF* (hepatocyte growth factor) with suggestive association to TEW, showed a trend toward significance in the replication phase (OR [95% CI] = 1.11 [0.99–1.26], *P* = .071) (Table III) and was only associated with TEW (see Table E8 in the Online Repository available at www.jacionline.org).

### Analysis of known asthma variants

In total, 207 independent SNPs were used to characterize genetic similarity of the wheeze phenotypes to the known genetics of asthma. In total, 86 of 207 SNPs were nominally associated (*P* < .05) with the same direction of effect, such that the risk allele for asthma was also associated with a higher risk of wheeze. Nine SNPs passing nominal significance showed the opposite direction of effect (see Table E9 in the Online Repository available at www.jacionline.org).

**FIG 1.**
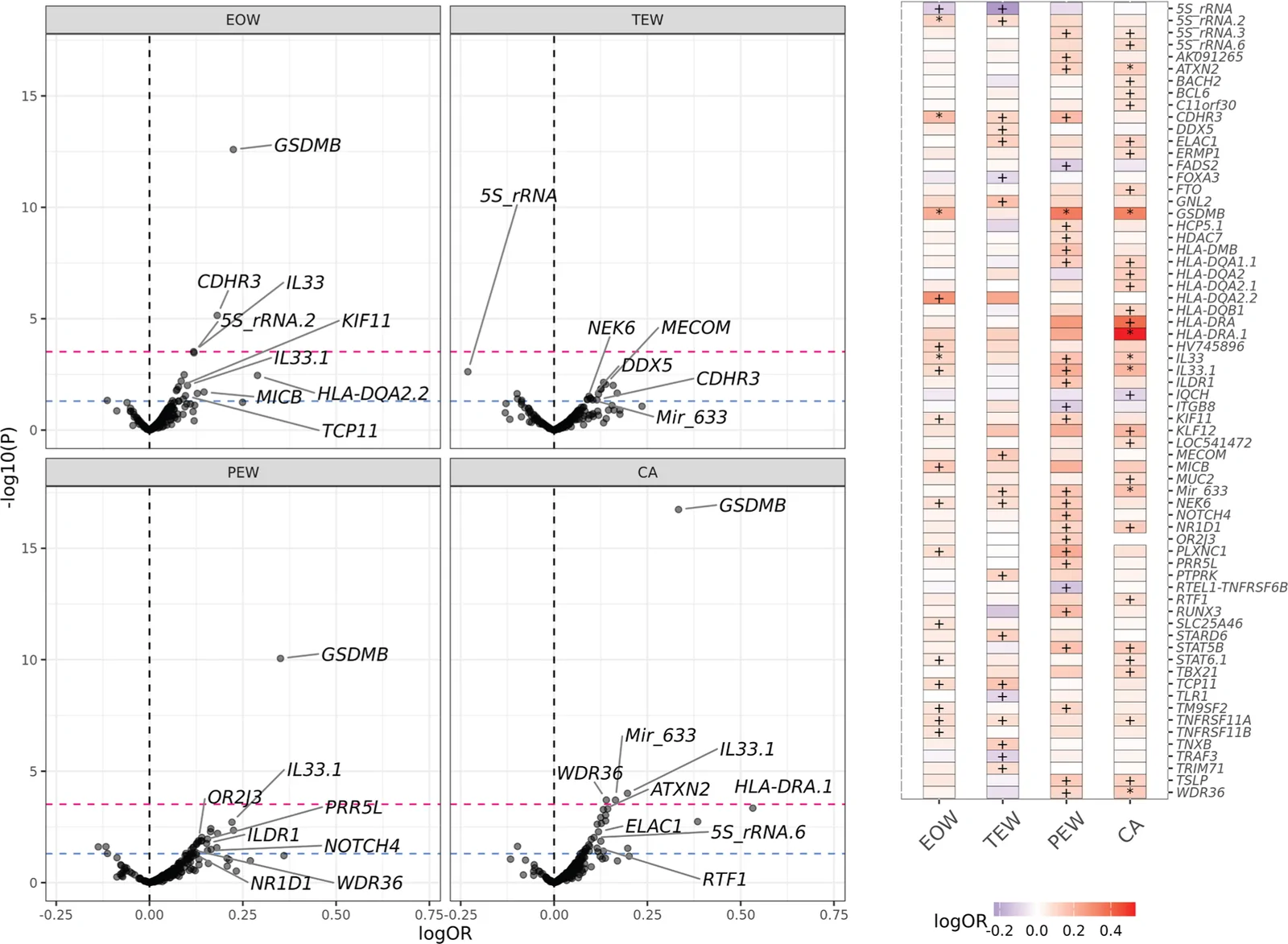
*Left,* Volcano plots of 4 wheeze phenotypes: EOW, TEW, PEW, and CA (*top* to *bottom*). *Pink dashed line* indicates FDR < 0.05; *blue dashed line,* nominal significance level (*P* < .05). Plots show if there is trend in risk effects of known asthma variants and effect in wheeze, with log odds for wheeze status on x-axis and *–*log_10_(*P* value) on y-axis. *Right,* Variants with nominally significant association in at least one phenotype. Cells are colored according to logOR; *stars* indicate level of significance (+*P* < .05, *FDR < 0.05).

Most of the significant associations with the same direction of effect were seen for EOW, PEW, and CA (Fig 1). Four loci passed correction for multiple testing (FDR < 0.05) with directional consistency in EOW, namely *GSDMB* (gasdermin B), *CDHR3, IL33,* and *5S-rRNA*. Only *GSDMB* passed FDR in PEW. Last, 7 loci passed FDR in CA, specifically *ATXN2, GSDMB, HLA-DRA,* both *IL33* loci, *Mir633,* and *WDR36.* For TEW, limited directional replication was present (ρ = 0.03, *P* = .604), and no loci passed FDR. *CDHR3* was the only locus with a positive association with TEW (rs6967330-A, OR = 1.12, *P* = .044), and had previously been associated with preschool asthma.^9^

### Genetic similarity between adult traits and preschool wheeze

**FIG 2.**
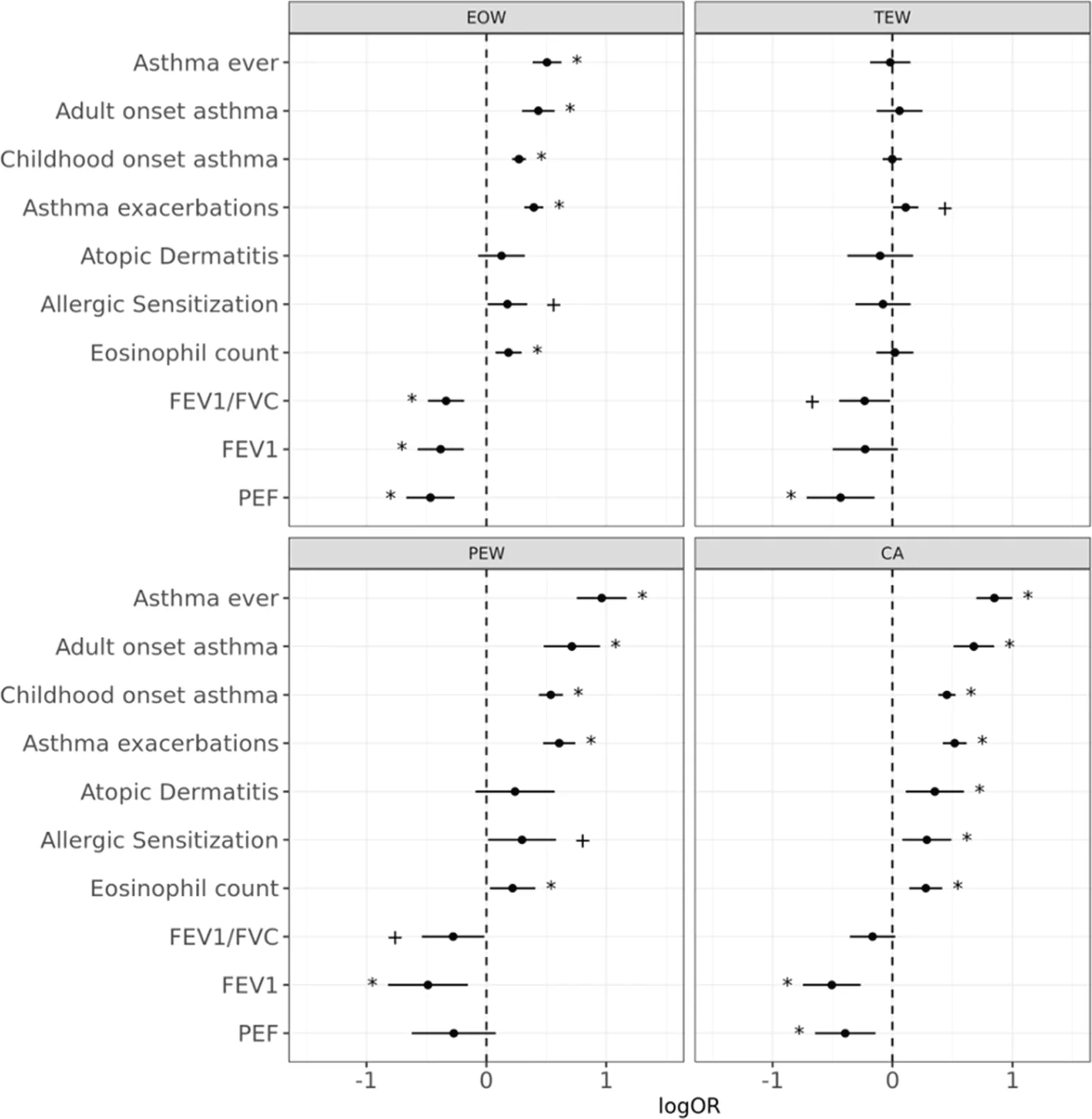
Log odds ratios increase for wheeze risk of shared genetic influence between adult traits and preschool wheeze. Association results of variant coefficients between adult traits (y-axis) and preschool wheeze (boxes), including 95% confidence intervals for association result. *Stars* indicate significance of association (+*P* < .05, *FDR < 0.05).

EOW significantly overlapped with most of the asthma-related traits, including asthma at older ages, eosinophil count, allergic sensitization, and lung function. A similar pattern of association was seen for PEW, with significant overlap with 6 of 10 traits, and CA with overlap for 9 of 10 traits. TEW showed a distinct association pattern compared to the other phenotypes, and only showed significant overlap with peak expiratory flow (PEF) (OR [95% CI] = 0.64 [0.48–0.86], *P* = 2.73e-3). Forced expiratory volume in 1 second (FEV_1_)/forced vital capacity (FVC) ratio (0.79 [0.64–0.98], *P* = .032) and asthma exacerbations (1.12 [1.004–1.24], *P* = .041) showed nominally significant overlap with TEW (Fig 2). Results are shown in Tables E10 and E11, and scatter plots of SNP effect sizes between traits and preschool wheeze are shown in Figs E3-E13, all of which are in the Online Repository available at www.jacionline.org.

### Lung development genes

When only considering variants closest to genes associated with lung development, the number of statistical tests was reduced from ;3.1M to ;10K. The variant rs2214823 near the *HGF* gene was significantly associated with TEW after correcting for multiple testing (see Figs E14 and E15 in the Online Repository available at www.jacionline.org). No other signals were detected across the 4 phenotypes. The TEW risk allele of rs2214823 was associated with a lower PEF in adults (*P* = 6.86e-03), but not FEV_1_ or FEV_1_/FVC (see Table E12 in the Online Repository).

## DISCUSSION

Wheeze in preschool age, and particularly in the first 3 years of life, is a common clinical syndrome that is still poorly understood. It clearly represents heterogeneous mechanisms, as also indicated by the different temporal subtypes, with a large proportion of children experiencing remission before age 6.^6,33^ Diagnosing and subtyping wheezing illnesses in the first 3 years of life is challenging both clinically and in a research context. The mechanistic understanding of this process remains a black box because of the limited lung function assessment options and the absence of markers of airway inflammation in this age group. For this reason, many clinicians and researchers are hesitant to diagnose asthma before age 3.

Our GWAS of recurrent wheeze or asthma before age 3 years (EOW) showed genetic similarity, with some of the traits also characterizing later asthma, including blood eosinophil count, allergic sensitization, and lower lung function. This indicates that some of the mechanisms involved in later asthma are also involved in wheeze in the first 3 years of life, including eosinophilic inflammation. However, even though there was an overlap between previously reported asthma loci and EOW-associated loci (Fig 1), the pattern of association was different for EOW compared to studies in older children and adults. The 17q locus showed much stronger association than the remaining asthma loci; the second strongest locus was *CDHR3,* which was not the case for childhood-onset asthma (before 12 years) or adult-onset asthma (onset between 26 and 60.5 years).^13^

The 17q locus (at chromosome 17q12–21) is one of the strongest and most robustly replicated asthma loci.^34^ It is specifically associated with childhood-onset asthma and increases the susceptibility to rhinovirus respiratory illnesses.^35^ The locus spans several genes, but recent evidence points toward *GSDMB* as the causal asthma gene in the region.^36^ *GSDMB* is involved in a form of programmed cell death called *pyroptosis,* resulting in excessive airway inflammation after triggers such as viral infections.^37^ *CDHR3* was discovered in a GWAS of asthma with severe exacerbations at age 2–6 years,^9^ and it was later demonstrated that CDHR3 acts as a rhinovirus C receptor^38^ and increases the risk of rhinovirus C respiratory illnesses in the first 3 years of life.^39^ Interestingly, there is interaction between 17q and *CDHR3* risk variants, so that the *CDHR3* risk variant only increases the risk for severe asthma exacerbations in individuals carrying at least one 17q risk allele.^40^ This indicates a common mechanism for these two loci, potentially involving an exaggerated inflammatory response to viral infections, and on the basis of our genetic findings, this mechanism seems to be particularly important in the pathogenesis of EOW.

Our two subtypes of early-onset wheeze, PEW and TEW, differed in their pattern of genetic associations. PEW showed similarity in genetic risk with asthma, eosinophil counts, and low lung function and showed associations to previously reported asthma genes. In contrast, TEW was mainly influenced by risk of low PEF and possibly also FEV_1_/FVC and preschool asthma exacerbations. These results suggest that distinct genetic mechanisms underlie TEW compared to PEW, although we cannot exclude the notion that lack of statistical power due to low heritability can play a role.

Several nongenetic studies have compared TEW and PEW in terms of associated traits. The Tucson Children’s Respiratory Study (n = 125) found that TEW was associated with lower lung function shortly after birth, while PEW had normal lung function at birth and lower lung function at age 6.^6^ In contrast, the Perth birth cohort study (n = 243)^41^ reported reduced infant lung function in children with PEW but not TEW, while COPSAC_2000_, with higher statistical power (n = 403), found that persistent wheeze was associated with reduced lung function already at birth, with further reduction to age 7 years, and transient wheeze showed a trend of reduced lung function at birth.^42^ The large ALSPAC study (n = 6265) reported reduced lung function at age 7 for both transient and persistent wheeze.^33^ Regarding atopic traits, most studies have shown that persistent (but not transient) wheeze is associated with atopic traits such as allergic sensitization.^6,33^ The literature thus suggests that wheeze restricted to the first 3 years of life is associated with reduced lung development and early lung function decline, while wheeze persisting after 3 years is more atopic in nature, but that both etiologies involve reduced lung function by the age of 6, possibly through different mechanisms. Our analysis of shared genetic influence supports these findings by showing that genetic risk for low PEF and possibly low FEV_1_/FVC and asthma exacerbations are associated with TEW, while PEW and CA were consistent with genetic risk for asthma at older ages, atopy and lung function. In line with our results, a study from the PIAMA cohort showed that TEW was associated with known chronic obstructive pulmonary disease genes.^43^ Overall, this suggests that TEW is distinct from childhood asthma and more likely involves impaired lung development, and as a result impaired respiratory function.

We did not identify novel susceptibility loci at the genome-wide association level in our study. A recent GWAS of temporal childhood wheeze phenotypes was performed in 9658 subjects from the STELAR and UNICORN cohorts. This study utilized latent class analysis–derived temporal phenotypes based on = time points to define wheeze trajectories from birth to 18 years and identified one novel genome-wide significant variant near *ANXA1* associated with wheeze persisting from birth until adolescence.^16^ The identified *ANXA1* variant was not present in our study, and we had no strong tagging variant, potentially due to the low allele frequency (~2%). We were therefore unable to test if this variant replicated in our study.

In the replication phase, a single variant rs2214823 near *HGF* showed a trend toward replication. The *HGF* variant also showed significant association to TEW after adjustment for multiple testing when considering only genes involved in fetal lung development. No associations were found between the *HGF* variant and EOW, PEW, or CA. Additionally, we found that the variant was associated with PEF in the UK Biobank (ukbiobank.ac.uk), indicating a possible role in adult lung function. Even though replication failed for rs2214823, these results suggest that TEW may be predisposed to wheeze in early childhood as a result of underdeveloped airways, a view shared by Martinez et al.^6^ HGF has been implicated in pulmonary fibrosis^44^ and is also expressed in embryonic lung tissues,^45,46^ suggesting it may play a role in both tissue repair and lung development.

We found that the 17q12–21 locus was strongly associated with EOW, PEW, and CA but not TEW, despite the relatively large sample size in the discovery phase. In contrast to our findings, a study combining multiethnic US birth cohorts (CREW) found associations between multiple 17q variants and latent class analysis–derived transient wheeze from 0 to 11 years.^47^ In the present study, we used a different approach to define transient wheeze based on children who wheezed more than 3 times or were diagnosed with asthma before age 3. Simultaneously, they were completely free of wheeze and asthma at age 6. This contrasts with the definition in the CREW study, in which ~20% wheezed at age 6, meaning that their transient phenotype could potentially overlap with our persistent phenotype. In line with our findings, a study combining UK birth cohorts (STELAR) found no association between transient wheeze and 17q in 7719 children.^48^ It should be noted that some cohorts in the two studies described above participated in the discovery phase of this study.

In our study, *CDHR3* was associated with both persistent (OR = 1.17, *P* = 6.2e-3) and transient wheeze (OR = 1.12, *P* = .044). The STELAR study found that *CDHR3* was associated with persistent and intermittent wheeze, but not with transient and late-onset wheeze. This contradicts our findings, but our definition of TEW differed slightly from that of STELAR in that we required at least 3 episodes of wheeze before the age of 3 to classify TEW, in contrast to a single episode in STELAR. The higher symptom burden of TEW in this study may explain the different results.

This study represents what is to our knowledge the largest GWAS of preschool wheeze phenotypes to date, providing a resource for understanding the genetic background of wheezing and asthma-like symptoms in preschool children. Another study strength is our inclusion of a large number of well-characterized birth cohorts with wheeze phenotypes characterized longitudinally, thereby minimizing the risk of recall bias.

Preschool wheeze is a heterogenous and poorly defined syndrome. To be able to perform a sufficiently powered GWAS, we used all available cohorts with genotype and phenotype data, and we standardized the criteria for defining wheeze/asthma as much as possible. This resulted in similar but not uniform definitions, as shown in Table E2. Lung function assessments in preschool age is difficult, and few cohorts have information on reversible airflow limitation in this age group. In order to achieve statistical power for a GWAS, we therefore had to rely on wheeze symptoms observed by parents and/or physicians as indirect measures of airflow limitation. Similarly, we did not verify the asthma diagnosis at 5 to 7 years via spirometry because only some children could perform a reliable spirometry maneuver in this age, and often a single baseline measurement would be an insufficient basis for a diagnosis. It is another limitation of our study that we did not include a late-onset wheeze phenotype. This decision was made because of the expected low numbers of children with this phenotype, which therefore limited statistical power during data analysis. Also, in spite of being the largest GWAS on preschool wheeze to date, the sample sizes were still relatively small compared to previous GWAS using other definitions of asthma, which is also reflected in the paucity of genome-wide significant associations. Given the limited statistical power in our discovery analyses, we were not able to estimate genetic correlation with other traits using the standard approach of LD score regression. LD score regression is more robust than our approach because LD structure is taken into account.^24^

The strength of GWAS is the unbiased approach to identifying mechanisms involved in the disease pathogenesis as future therapeutic or preventive targets. However, genetic variants on their own are generally weak and have limited predictive potential, as demonstrated in previous asthma GWASs.^18^ Adding exposure data may lead to more precise risk estimates, and improved prediction might also be obtained in the future using more downstream and dynamic biomarkers, such as gene expression, methylation, and protein levels. Our genetic findings indicate that GSDMB and CDHR3-related pathways are promising targets for improved treatment and prevention of early-onset wheeze and asthma.

In conclusion, we performed what is to date the largest GWAS on recurrent wheeze or asthma in the first 3 years of life and found the strongest signals for the 17q and *CDHR3* loci, both of which are implicated in responses to respiratory infections. This phenotype also showed genetic similarity with asthma at older ages and asthma-related traits in terms of blood eosinophils, allergic sensitization, and low lung function. The genetic similarity with atopic traits was driven by children with symptoms persisting to age 6, while children with symptoms that remitted before age 6 only showed similarity to genetics of reduced lung function in adulthood and risk of early-life asthma exacerbations. These results suggest that TEW is influenced by other molecular mechanisms than persistent phenotypes. Future studies should investigate the genetic mechanisms and biomarkers of early-onset wheeze to both improve prediction of the disease course and improve disease treatment.

## Supplementary Material

1

2

3

4

5

6

7

8

9

## Figures and Tables

**TABLE I.** Number of cases and controls for phenotype by cohort

	EOW		TEW		PEW		CA	
Cohort	Cases	Controls	Cases	Controls	Cases	Controls	Cases	Controls
Discovery								
ALSPAC	1272	4667	496	5193	338	5873	682	5020
BAMSE	141	341	62	420	65	417	236	246
COPSAC_2000_	61	255	15	303	48	288	45	262
DNBC	103	850	42	850	12	850	32	850
EGEA	203	1632	—	—	—	—	—	
GENR	667	2294	383	1985	109	2259	286	3089
INMA	163	644	91	648	50	692	139	636
LISA/GINI	74	963	46	1005	27	1100	118	1009
MAAS	294	568	151	545	143	719	166	696
MOBA	88	528	—	—	—	—	—	—
PIAMA	125	252	52	317	55	306	62	317
RAINE	82	1116	—	—	—	—	—	—
MAGICS	—	—	—	—	130	397	284	397
B58C T1DGC	—	—	—	—	—	—	40	2215
B58C WTCC	—	—	—	—	—	—	14	1319
Total	3273	14,110	1338	11,266	977	12,901	2104	12,970
Replication								
SWS	358	846	131	589	94	626	246	492
CAPPS/SAGE	81	182	50	149	21	149	49	201
NTR	150	1886	34	1992	112	1914	—	—
COAST	—	—	14	187	—	—	—	—
COPSAC_2010_	124	555	88	559	30	617	146	501
SEATON	81	492	35	533	46	522	80	451
Isle of Wight	161	847	68	917	65	920	179	768
Total	1235	6120	734	6589	471	6171	725	3928

Dashes indicate that it was not possible to define all phenotypes in every cohort.

**TABLE II.** Association of 17q12 locus represented by rs7219923 across wheeze phenotypes

Phenotype	Nearest gene	SNP	EA/NEA	OR	95% CI	*P* value	No.
EOW	*GSDMB*	rs7219923	T/C	1.24	1.173–1.311	3.05E-14	17,383
TEW				1.063	0.979–1.153	.1423	12,604
PEW				1.369	1.237–1.516	1.28E-09	13,878
CA				1.335	1.241–1.438	1.33E-14	15,074

**TABLE III.** Association results containing novel variants carried forward to replication stage

			Discovery				Replication				Discovery + replication		
Phenotype	SNP	Nearby gene	OR (95% CI)	*P*	N	*P*_het_	OR (95% CI)	*P*	N	*P*_het_	OR (95% CI)	*P*	Direction
TEW	Chr7 rs2214823-A	*HGF*	1.25 (1.15–1.35)	2.32E-07	12, 604	.116	1.11 (0.99–1.26)	.071	7, 323	.066	1.20 (1.12–1.29)	1.52E-07	++
EOW	Chr18 rs11877254-A	*DCC*	1.16 (1.10–1.23)	2.33E-07	17, 383	.933	0.95 (0.85–1.04)	.37	7, 355	.307	1.13 (1.07–1.18)	3.93E-06	+–
PEW	Chr6 rs6941858-G	*ARG1*	1.75 (1.41–2.17)	3.48E-07	13, 878	.069	0.72 (0.38–1.38)	.33	6, 642	.325	1.38 (1.24–1.49)	6.22E-06	+–
CA	Chr9 rs12554253-T	*PRUNE2*	1.35 (1.20–1.51)	3.97E-07	15, 074	.749	1.03 (0.84–1.26)	.76	4, 653	.761	1.26 (1.14–1.39)	5.30E-06	++
EOW	Chr12 rs12817967-A	*RPL31P10*	1.15 (1.09–1.22)	8.75E-07	17, 383	.012	1.00 (0.91. 1.10)	.93	7, 355	.934	1.09 (1.04–1.15)	8.26E-04	++
EOW	Chr15 rs1896796-A	*ADAMTSL3*	1.15 (1.09–1.22)	1.22E-06	17, 383	.993	1.01 (0.92–1.11)	.79	7, 355	.793	1.11 (1.06–1, 17)	3.91E-05	++
CA	Chr10 rs12767108-A	*SLC29A3*	1.29 (1.17–1.44)	1.36E-06	15, 074	.954	1.06 (0.89–1.27)	.5	4, 653	.504	1.23 (1.12–1.35)	6.87E-06	++
PEW	Chr1 rs3752543-C	*CAMTA1*	1.37 (1.21–1.56)	1.71E-06	13, 878	.102	1.00 (0.81–1.23)	.99	6, 642	.998	1.20 (1.11–1.29)	4.90E-05	++
EOW	Chr1 rs12082330-G	*SLC35D1*	1.30 (1.17–1.45)	1.74E-06	17, 383	.479	0.97 (0.82–4.46)	.8	7, 355	.796	1.16 (1.07–1.23)	4.30E-04	+–
TEW	Chr6 rs505000-C	*SLC22A2*	1.27 (1.15–1.40)	3.29E-06	12, 604	.711	1.10 (0.93–1.29)	.25	7, 323	.098	1.14 (1.06–1.21)	8.18E-04	++

Results from both phases are shown for allele conferring risk in discovery phase. *P*_het_ indicates *P* value for heterogeneity test across included cohorts.

## Clinical implication:

Preschool wheeze phenotypes displayed distinct patterns of single SNP associations and genetic enrichment with asthma-related traits, indicating different etiologies.

## Data-sharing statement:

The COPSAC biobank is publicly available at the Danish National Biobank (www.biobankdenmark.dk), and data will become available in the Danish Data Archive (www.sa.dk) upon request to the corresponding author.

## Abbreviations used

CA: Current asthma
CDHR3: Cadherin-related family member 3
CI: Confidence interval
EAGLE: EArly Genetics and Lifecourse Epidemiology
EOW: Early-onset wheeze
FDR: False discovery rate
FEV_1_: Forced expiratory volume in 1 second
FVC: Forced vital capacity
GABRIEL: Multidisciplinary Study to Identify the Genetic and Environmental Causes of Asthma in the European Community
GSDMB: Gasdermin B
GSMR: Generalised Summary-data–based Mendelian Randomisation
GWAS: Genome-wide association study
HGF: Hepatocyte growth factor
LD: Linkage disequilibrium
OR: Odds ratio
PEF: Peak expiratory flow
PEW: Persistent early wheeze
SNP: Single nucleotide polymorphism
TEW: Transient early wheeze
