# Supplementary material for: Genetic characterization of preschool wheeze phenotypes
Source: J Allergy Clin Immunol. Author manuscript; Available in PMC 2026 Sep 21. (PMC13592397; doi:10.1016/j.jaci.2025.07.015)
Supplement: Supplementary file 1 [file NIHMS2180477-supplement-1.docx]

# **Supplementary figures**

**Supplementary figure 1.** Association results of variants to the wheezing phenotypes. Pink solid line is the FDR line and blue-dashed is the nominal significance level.

 **Supplementary figure 2.** QQ-plots of the genome-wide association analysis, showing the expected vs. observed p-value distributions in the analysis.

**Supplementary figures 3-13.** XY plots of SNP effects on related traits vs. SNP effects on wheeze phenotypes. Contains information on included SNPs, and their effects in related traits and investigated phenotypes as well as the closest gene. Black: P < 0.05 in wheeze GWAS. Grey: P > 0.05 in wheeze GWAS.

Supplementary figure 14. Association results of variants located within 10 kb from genes known to be differentially expressed during fetal lung development. Pink solid line is the FDR line and blue-dashed is the nominal significance level.

 **Supplementary figure 15. QQ-plots of the genome-wide association analysis of lung development genes, showing the expected vs. observed p-value distributions in the analysis.**
