# Supplementary material for: Genetic characterization of preschool wheeze phenotypes
Source: J Allergy Clin Immunol. Author manuscript; Available in PMC 2026 Sep 21. (PMC13592397; doi:10.1016/j.jaci.2025.07.015)
Supplement: Supplementary file 2 [file NIHMS2180477-supplement-2.docx]

# **Supplementary tables**

**Supplementary table 1. This table contains an overview of the phenotype definitions used in this study.**

**Supplementary table 2. The population used in this study comes from the EAGLE consortium which aims to understand how early childhood traits affect disease and well-being in later life. The Gabriel Project also constitutes a substantial part of the included studies and this comprises one of the biggest genetic studies of asthma, genotyping 10,365 individuals with asthma diagnosis and 16,110 unaffected. This table contains all basic characteristics from all included studies in the discovery phase.**

**See DiscoveryCohortCharacteristics.xlsx**

**Supplementary table 3. This table contains all basic characteristics from all included studies in the replication phase.**

**See ReplicationCohortCharacteristics.xlsx**

**Supplementary table 4. Heritability of the four wheeze phenotypes calculated with LD-score regression. Heritability is shown in the top row, followed by the lambda GC, mean chi square statistic and regression intercept.**

**Supplementary table 5. Top associated variants in 17q-21 locus across the four phenotypes.**

**Supplementary table 6. All variants in the wheeze GWAS, which showed at least suggestive association (P < 5e-5), with r2 < 0.01 in a 1 Mb window.**

**See TopLoci.xlsx**

**Supplementary table 7. This table contains the number of additional variants imputed from the summary stats. Each phenotype is represented by a row, containing information the number of SNPs included in the study (“N SNPs in study”), minimum number of individuals carrying this SNP before imputing (“Minimum N 90th percentile”), how many SNPs were included (“Included SNPs for imputation”), how many SNPs were imputed and how many total SNPs were included in the study.**

**Supplementary table 8. For each phenotype, here is displayed the number of cases and controls and the number of asthma variant with significant associations to wheeze (P < 0.05) or FDR corrected associations (FDR < 0.05), and those with the same direction of effect as the risk allele in the included asthma GWAS (“Same” column). We also calculated Spearman’s correlation between all log-odds ratios regardless of p-value.**

**Supplementary table 9. All candidate variants showing nominal significance (P < 0.05) in the wheeze phenotypes. They are all mapped to the closest gene.**

**See NominalCandidateGenes.txt**

**Supplementary table 10. Results of the genetic overlap analysis, for each trait and phenotype. Beta indicates the effect size of the overlap, and the standard error and p-value is associated with this. The column n.snps indicates the number of SNPs included in the analysis, which are all SNPs in a window of 1 Mb with an r2 < 0.05. GSMR allows for residual correlation.**

**See GeneticOverlapResults.xlsx**

**Supplementary table 11. Overview of the SNPs including the in the genetic overlap analysis. The wheeze effect is shown in Z-scores since the FIZI-imputed summary results are used in this analysis to increase probability of overlap with the related traits.**

**See GeneticOverlapSNPs.xlsx**

**Supplementary table 12. Results of associations between the HGF-variant and lung function measurements relevant for obstructive disease in UK Biobank.**
