# Supplementary material for: Genetic characterization of preschool wheeze phenotypes
Source: J Allergy Clin Immunol. Author manuscript; Available in PMC 2026 Sep 21. (PMC13592397; doi:10.1016/j.jaci.2025.07.015)
Supplement: Supplementary file 3 [file NIHMS2180477-supplement-3.docx]

**Supplementary material**

# Genetic characterization of preschool wheeze phenotypes

Kasper Fischer-Rasmussen, MSc^1^, Raquel Granell, PhD^3^, Anders Ulrik Eliasen, Phd^1^, Eskil Kreiner, MD, Phd^1^, Casper-Emil Tingskov Pedersen, Phd^1^, Yang Luo, Phd^1^, Bo Chawes, MD, DMSc^1^, Jakob Stokholm, MD, Phd^1^, Ann-Marie Malby Schoos, MD, Phd, DMSc^1^, Ashish Kumar, Phd^4,5^, Anne-Marie Nybo Andersen, MD, Phd^6^, Bjarke Feenstra, Phd^6^, Frank Geller, Phd^6^, Valérie Siroux, Phd^7^, Florence Demenais, MD^7^, Emmanuelle Bouzigon, MD, Phd^7^, Vincent Jaddoe, Phd^8,9^, Ralf van der Valk, Phd^8,9^, Liesbeth Duijts, MD, Phd^8,9,10^, Jordi Sunyer, MD, Phd^11,12,13^, Mónica Guxens, MD, Phd^11,12,13,14^, Marcella Marinelli, Phd^12,15^, Mariona Bustamante, Phd^12,16^, Joachim Heinrich, Phd^17,18^, Marie Standl, Phd, John Curtin, MD, Phd^20,21^, Angela Simpson, MD, Phd^21^, Clare Murray, MD, Phd^21^, Bo Jacobsson, MD, Phd^22,23^, Ronny Myhre, Phd^22^, Craig E Pennell ,MD, Phd^24,25,26,27^, Denise Daley, MD, Phd^28^, Carole Ober, Phd^29^, James E. Gern, MD^30^, Daniel Jackson, MD^30^, Dorret I. Boomsma, Phd^31^, Jouke-Jan Hottenga, Phd^31^, Abdel Abdellaoui, Phd^31^, John W Holloway, Phd^32,33^, Sam Collins, MD^32,34^, Stephen Turner, MD^35,36^, S Hasan Arshad, MD, Phd^32,33,37^, Anhar Ullah, Phd^38^, Erik Melén, MD, Phd^4,5^, John Henderson, MD, FRCP, FRCPCH, FRCPEd^3†^, Hans Bisgaard, MD, DMSc^1†^, Anders Gorm Pedersen, MSc, Phd^2^, Adnan Custovic, MD, Phd, FMedSci^39^, Judith M. Vonk, Phd^40^, Gerard H. Koppelman, MD, Phd^40,41^, Michael Kabesch, MD^42^, Klaus Bønnelykke, MD, Phd^1^, GABRIEL consortium, [the EArly Genetics and Lifecourse Epidemiology (EAGLE) Consortium](https://www.nature.com/articles/ng.2694#group-2)

**Cohort recruitment details, acknowledgements and funding statements**	**3**

ALSPAC	3

B58C (British 1958 birth cohort)	5

BAMSE	6

CAPPS/SAGE	7

COAST	9

COPSAC2000	10

COPSAC2010	10

DNBC	11

EGEA	13

GENERATION R	14

GINI/LISA	16

IoW	18

INMA	19

MAAS	20

ISAAC II / MAGICS	22

NTR	23

RAINE	24

PIAMA	25

MOBA	27

SWS	29

SEATON	30

**Imputation of GWAS summary statistics**	**32**

**Data Visualization**	**32**

**Genetic Similarity Between Adult Traits and Preschool wheeze**	**33**

**Published GWAS on Asthma and Asthma-related Traits in Adulthood**	**33**

Asthma ever in UK Biobank	34

Childhood vs. adult onset asthma in UK BIobank	34

Asthma exacerbations (0-6 year)	35

Atopic dermatitis	36

Allergic sensitization	36

Eosinophil count	36

Lung function measurements	37

**Opposite Direction of Effects: Analysis of Known Asthma Variants**	**37**

**Supplementary figures**	**38**

**Supplementary tables**	**52**

**Supplementary code**	**59**

# **Cohort recruitment details, acknowledgements and funding statements**

## ALSPAC

**Recruitment**

Pregnant women resident in Avon, UK with expected dates of delivery between 1st April 1991 and 31st December 1992 were invited to take part in the study. 20,248 pregnancies have been identified as being eligible and the initial number of pregnancies enrolled was 14,541. Of the initial pregnancies, there was a total of 14,676 foetuses, resulting in 14,062 live births and 13,988 children who were alive at 1 year of age. When the oldest children were approximately 7 years of age, an attempt was made to bolster the initial sample with eligible cases who had failed to join the study originally. As a result, when considering variables collected from the age of seven onwards (and potentially abstracted from obstetric notes) there are data available for more than the 14,541 pregnancies mentioned above: The number of new pregnancies not in the initial sample (known as Phase I enrolment) that are currently represented in the released data and reflecting enrolment status at the age of 24 is 906, resulting in an additional 913 children being enrolled (456, 262 and 195 recruited during Phases II, III and IV respectively). The phases of enrolment are described in more detail in the cohort profile paper and its update (see footnote 5 below). The total sample size for analyses using any data collected after the age of seven is therefore 15,447 pregnancies, resulting in 15,658 foetuses. Of these 14,901 children were alive at 1 year of age [(1,2)](https://www.zotero.org/google-docs/?Byghp4).

Ethical approval for the study was obtained from the ALSPAC Ethics and Law Committee and the Local Research Ethics Committees. Informed consent for the use of data collected via questionnaires and clinics was obtained from participants following the recommendations of the ALSPAC Ethics and Law Committee at the time.

Please note that the study website contains details of all the data that is available through a fully searchable data dictionary and variable search tool: [Explore data and samples | Avon Longitudinal Study of Parents and Children | University of Bristol](http://www.bristol.ac.uk/alspac/researchers/our-data/)

**Acknowledgements and Funding**

We are extremely grateful to all the families who took part in this study, the midwives for their help in recruiting them, and the whole ALSPAC team, which includes interviewers, computer and laboratory technicians, clerical workers, research scientists, volunteers, managers, receptionists and nurses.The UK Medical Research Council and Wellcome (Grant ref: 217065/Z/19/Z) and the University of Bristol provide core support for ALSPAC. GWAS data was generated by Sample Logistics and Genotyping Facilities at the Wellcome Trust Sanger Institute and LabCorp (Laboratory Corporation of America) using support from 23andMe. FLG genotyping was funded by an MRC centenary award (awarded to L Paternoster).

## B58C (British 1958 birth cohort)

**Recruitment**

The British 1958 birth cohort is an ongoing follow-up of all persons born in England, Scotland and Wales during one week in 1958. At ages 7, 11, 16, 23, 33 and 42 years, a history of hay fever or allergic rhinitis was obtained by interview with a parent (aged 7-16) or the cohort member (ages 23-42), At the age of 44-45 years, the cohort were followed up with a biomedical examination and blood sampling, from which a DNA collection was established as a nationally representative reference panel, and the presence of circulating IgE antibodies to house dust mite, mixed grass pollen and cat fur was ascertained by Hytec enzyme immunoassay, with a detection threshold of 0.35 kU/L.

**Acknowledgements and Funding**

We acknowledge use of phenotype and genotype data from the British 1958 Birth Cohort DNA collection, funded by the Medical Research Council grant G0000934 and the Wellcome Trust grant 068545/Z/02. Genotyping for the B58C-WTCCC subset was funded by the Wellcome Trust grant 076113/B/04/Z. The B58C-T1DGC genotyping utilized resources provided by the Type 1 Diabetes Genetics Consortium, a collaborative clinical study sponsored by the National Institute of Diabetes and Digestive and Kidney Diseases (NIDDK), National Institute of Allergy and Infectious Diseases (NIAID), National Human Genome Research Institute (NHGRI), National Institute of Child Health and Human Development (NICHD), and Juvenile Diabetes Research Foundation International (JDRF) and supported by U01 DK062418. B58C-T1DGC GWAS data were deposited by the Diabetes and Inflammation Laboratory, Cambridge Institute for Medical Research (CIMR), University of Cambridge, which is funded by Juvenile Diabetes Research Foundation International, the Wellcome Trust and the National Institute for Health Research Cambridge Biomedical Research Centre; the CIMR is in receipt of a Wellcome Trust Strategic Award (079895). The B58C-GABRIEL genotyping was supported by a contract from the European Commission Framework Programme 6 (018996) and grants from the French Ministry of Research.

The funding agencies did not have any influence on design and conduct of the study; collection, management, and interpretation of the data; or preparation, review, or approval of the manuscript

## BAMSE

**Recruitment**

BAMSE is a Swedish birth cohort study. A total number of 4,089 newborn infants were recruited between 1994 and 1996 in the Stockholm area^19^. The first questionnaire data, dealing with parental allergic diseases, socio-economic status and residential characteristics, was obtained when the children were about 2 months. Similar questionnaires, with a focus on the children’s symptoms related to asthma and allergic diseases including eczema, were answered by the parents when the children were approximately 1, 2, 4, 8 and 12 years old. At 8 years of age, all children were invited to clinical testing, and blood samples were obtained from 2,480 children (~60%). DNA was extracted from 2,033 samples after exclusion of samples with too little blood, lack of questionnaire data, or if parental consent to genetic analysis of the sample was not obtained. The imputation-related computational support was enabled by resources under “SNIC project sens2017589” provided by the National Academic Infrastructure for Supercomputing in Sweden (NAISS) at UPPMAX

**Acknowledgements and Funding**

BAMSE was supported by The Swedish Research Council, The Swedish Heart-Lung Foundation, Stockholm County Council (ALF) and the Strategic Research Programme (SFO) in Epidemiology at Karolinska Institutet. Genome-wide genotyping in BAMSE was funded by the European Commission as part of GABRIEL (A multidisciplinary study to identify the genetic and environmental causes of asthma in the European Community) contract number 018996 under the Integrated Program LSH-2004-1.2.5-1.

## CAPPS/SAGE

**Recruitment**

The Canadian Asthma Primary Prevention Study (CAPPS) was initiated in 1995 to assess the effectiveness of a multifaceted intervention program applied in the first twelve months of life on the primary prevention of asthma and other atopic disorders in high-risk infants (E6). Infants at high risk for developing asthma and other allergic disorders were identified and their mothers were recruited during their third trimester of pregnancy. Infants at high risk were defined as those who had a parent with asthma or two first-degree relatives with allergies or atopic disorders. The study had two recruitment centers in Canada (Vancouver and Winnipeg).

The Study of Asthma Genes and the Environment (SAGE), is derived from a populationbased cohort of 16,320 children born in province of Manitoba, Canada, between January 1, 1995 through December 31, 1995. A survey on child health and home environment exposures was mailed to the parents of this birth cohort in 2002. Following parent response to the mailed survey and approval to be contacted, a subset of children was invited to join the study at age 8-10 years. This included all children with parent-declared asthma and a comparable number of children without asthma, randomly selected following stratification of the received surveys by urban / rural location and family income. All recruited children underwent clinical assessment for asthma by a pediatric allergist (AB or JL) on the basis of the Canadian Asthma Consensus Guidelines.

**Acknowledgements and Funding**
The authors thank all families for their enthusiastic participation in this study. The authors also thank Diane Gagné, Muriel Grenon, and Dr. Paul Bégin for their invaluable participation in acquiring the subjects. Supported by the Canadian Institutes of Health Research and the Respiratory Health Network of the Fonds de la Recherche en Sante´ du Que´bec, and by AllerGen NCE, Inc., which paid for the genotyping. P.B. and K.T. are supported by the Allergy, Genes and Environment Network (AllerGen). T.J.H. is recipient of an Investigator Award from the Canadian Institutes of Health Research and a Clinician–Scientist Award in Translational Research from the Burroughs Wellcome Fund. D.D. is supported by grants from the Canadian Institutes of Health Research, Institutes of Gender and Health, Genetics, Population and Public Health, and the IMPACT fellowship, and is also supported by the Lung Association of British Columbia. C.L. is the chair-holder of the Canada Research Chair on genetic determinants in asthma and the director of the Genetics platform of the Respiratory Health Network of the Fonds de la Recherche en Sante´ du Que´bec, which financially supports the Saguenay–Lac-St.-Jean sample.

## COAST

**Recruitment**
The COAST longitudinal birth cohort study was designed to test for effects of immune development and early-life viral respiratory infections on the risk of allergic diseases and asthma ([9](https://www.atsjournals.org/doi/10.1164/rccm.201705-1021OC?url_ver=Z39.88-2003&rfr_id=ori:rid:crossref.org&rfr_dat=cr_pub%20%200pubmed)). Expectant families were recruited during the prenatal period. Entry criteria required that at least one parent have self-reported allergies or asthma. All babies were delivered in Madison area hospitals. Babies were excluded from the study if they were 36 weeks gestation or less, or if they developed, during the neonatal period, significant respiratory distress or other health conditions affecting either the lung or the immune system. The COAST study was approved by the University of Wisconsin–Madison Human Subjects Committee (2013-1044), and informed consent was obtained before enrollment. The study population included 289 at birth and 275 at age 3 years.

**Acknowledgements and Funding**

The authors thank the children and families of the COAST (Childhood Origins of Asthma Birth Cohort Study) cohort for their support and commitment to their studies, and acknowledge and appreciate the unique efforts of the COPSAC and COAST research teams. The Childhood Origins of Asthma Birth Cohort Study (COAST) is funded by NIH-NHLBI grant P01 HL070831.

## COPSAC2000

**Recruitment**

The COPSAC2000 birth cohort study is a prospective clinical study of a birth cohort of 411 infants born to mothers with a history of asthma. The newborns were enrolled at the age of 1 month, the recruitment of which was previously described in detail.[^25^](https://paperpile.com/c/OsX7sm/A3txq) The study was approved by the Ethics Committee for Copenhagen (KF 01- 289/96) and The Danish Data Protection Agency (2008-41-1754) and informed consent was obtained from both parents. For this study, only the parents were included.

**Acknowledgements and Funding**

COPSAC is funded by private and public research funds all listed on [www.copsac.com](http://www.copsac.com/). The Lundbeck Foundation (Grant no R16-A1694); The Danish Ministry of Health (Grant no 903516); Danish Council for Strategic Research (Grant no 0603-00280B); The Danish Council for Independent Research (Grant no 10-082884 and 271-08-0815); The Capital Region Research Foundation (No grant number) and NIH-NHLBI R01 HL129735 have provided core support for COPSAC.

## COPSAC2010

**Recruitment**
Pregnant women from eastern Denmark were invited during 2008–2010 to a novel unselected ‘COPSAC_2010_’ cohort. The women visited the clinic during pregnancy weeks 24 and 36. Their children were followed at the clinic with deep phenotyping and collection of biological samples at nine regular visits until the age of 3 and at acute symptoms. Randomized controlled trials of high‐dose vitamin D and fish oil supplements were conducted during pregnancy, and a trial of azithromycin for acute lung symptoms was conducted in the children with recurrent wheeze.

**Acknowledgements and Funding**
The authors gratefully express their gratitude to the children and families of the COPSAC cohort studies for all their support and commitment, and we acknowledge and appreciate the unique efforts of the COPSAC research team. COPSAC is funded by private and public research funds. The Lundbeck Foundation; the Pharmacy Foundation of 1991; Augustinus Foundation; the Danish Medical Research Council; and The Danish Pediatric Asthma Centre provided the core support for the study.

## DNBC

**Recruitment**

The DNBC consists of approximately 100,000 pregnancies enrolled via general practitioners from across Denmark in 1996–2003 [Olsen J, Melbye M, Olsen SF, Sørensen TI a., Aaby P, Nybo Andersen AM, et al. The Danish National Birth Cohort--its background, structure and aim.]. Mothers and their offspring have participated in multiple follow-ups, with the most recent 18 years after offspring birth. At the 11-year follow-up (2010–2014), approximately half of the caregivers (mostly mothers) responded to an online questionnaire regarding their child’s health and a wide range of questions regarding housing and indoor home environment at the time their child turned 11 years. At the 18-year follow-up, DNBC children were invited to reply to an online questionnaire in which questions on asthma diagnosis, medication and symptoms were asked. Individuals who had relevant data available from the 11-year and 18-year follow-ups were included in this study (Fig. [1](https://link.springer.com/article/10.1007/s10654-023-01051-y#Fig1)).

**Acknowledgements and Funding**

The Danish National Birth Cohort was established with a significant grant from the Danish National Research Foundation. Additional support was obtained from the Danish Regional Committees, the Pharmacy Foundation, the Egmont Foundation, the March of Dimes Birth Defects Foundation, the Health Foundation and other minor grants. The DNBC Biobank has been supported by the Novo Nordisk Foundation and the Lundbeck Foundation. Follow-up of mothers and children have been supported by the Danish Medical Research Council (SSVF 0646, 271-08-0839/06-066023, O602-01042B, 0602-02738B), the Lundbeck Foundation (195/04, R100-A9193), The Innovation Fund Denmark 0603-00294B (09-067124), the Nordea Foundation (02-2013-2014), Aarhus Ideas (AU R9-A959-13-S804), University of Copenhagen Strategic Grant (IFSV 2012), and the Danish Council for Independent Research (DFF – 4183-00594 and DFF—4183-00152).

## EGEA

**Recruitment**
The study population included 348 asthmatics and their family members and 416 control subjects. Probands (asthmatics and controls) aged 7 to 65 were ascertained in five French cities (Paris, Lyons, Marseilles, Montpellier, and Grenoble). All pro-bands and their two parents were born in France. In roughly 10,000 self-completed questionnaires (6,400 for potential cases and 3,600 for potential control subjects), 1,589 probands fulfilled inclusion criteria regarding place of birth and family structure (either proband with a spouse and at least one child, or proband with at least one sibling and two parents). A positive answer to four questions (Table 1) identified those with asthma. A total of 278 probands fulfilled this criterion. Seventy more who only answered positively to two or three questions were also included after examination of their medical record. Subjects with asthma comprised 213 adults and 135 children. Children control subjects were recruited in surgery hospitals. Two thirds of adult control subjects were population based (electoral rolls), and one-third were from surgery hospitals or check-up centers. Overall, half of the controls were population based.

**Acknowledgements and Funding**
The authors thank all the clinicians, interviewers, technicians, nurses, and secretaries who collected and coded the data and helped coordinate the study through the years. They also thank the subjects who participated in the study, without whose cooperation the study would not have been possible. Supported by INSERM/MSD-Chibret Convention, INSERM Networks for Clinical Research Grant 489012 and Public Health Research Grant 4934009.

## GENERATION R

**Recruitment**

The Generation R Study is a population-based prospective cohort study of pregnant women and their children from fetal life onwards in Rotterdam, The Netherlands. All children were born between April 2002 and January 2006, and currently followed until young adulthood. Of all eligible children in the study area, 61% were participating in the study at birth. Cord blood samples including DNA have been collected at birth. The study protocol was approved by the Medical Ethical Committee of the Erasmus Medical Centre, Rotterdam (MEC 217.595/2002/20). Written informed consent was obtained from parents of all participants.

**Acknowledgements and Funding**

The Generation R Study gratefully acknowledges the contributions of the children and their parents, the general practitioners, the hospitals and the midwives and pharmacies in Rotterdam. We would like to thank A. Abuseiris, K. Estrada, Dr. T. A. Knoch, and R. de Graaf as well as their institutions Biophysical Genomics, Erasmus MC Rotterdam, The Netherlands, and especially the national German MediGRID and Services@MediGRID part of the German D-Grid, both funded by the German Bundesministerium fuer Forschung und Technology under grants #01 AK 803 A-H and # 01 IG 07015 G, for access to their grid resources. We thank M. Jhamai, M. Ganesh, P. Arp, M. Verkerk, L. Herrera and M. Peters for their help in creating, managing and performing quality control for the genetic database. Also, we thank K. Estrada and C. Medina-Gomez for their support in the creation and analysis of imputed data. The Generation R Study is conducted by the Erasmus Medical Center in close collaboration with the School of Law and the Faculty of Social Sciences of Erasmus University Rotterdam, the Municipal Health Service, Rotterdam area, the Rotterdam Homecare Foundation and the Stichting Trombosedienst & Artsenlaboratorium Rijnmond (STAR-MDC; Rotterdam). The generation and management of genotype data for the Generation R Study were performed at the Genetic Laboratory of the Department of Internal Medicine at Erasmus Medical Center. The Generation R Study is made possible by financial support from the Erasmus Medical Center (Rotterdam), the Erasmus University Rotterdam and the Netherlands Organization for Health Research and Development (ZonMw; 21000074). The project received funding from the European Union's Horizon 2020 research and innovation programme (LIFECYCLE project, no 733206, 2016, EUCAN-Connect, project no. 824989, 2019; ATHLETE project, no. 874583, 2020). Dr Vincent Jaddoe received an additional grant from the Netherlands Organization for Health Research and Development (VIDI 016.136.361) and the European Research Council (Consolidator Grant, ERC-2014-CoG-648916). Dr Liesbeth Duijts received an additional grant from European Union's Horizon 2020 research and innovation programme (ENDOMIX project, no. 101136566, 2024). The researchers are independent from the funders. The study sponsors had no role in the study design, data analysis, interpretation of data, or writing of this report.

## GINI/LISA

**Recruitment**

The influence of Life-style factors on the development of the Immune System and Allergies in East and West Germany PLUS the influence of traffic emissions and genetics (LISAplus) Study is a population based birth cohort study. A total of 3094 healthy, full-term neonates were recruited between 1997 and 1999 in Munich, Leipzig, Wesel and Bad Honnef. The participants were not preselected based on family history of allergic diseases.[^39^](https://paperpile.com/c/OsX7sm/mxHXo) A total of 5991 mothers and their newborns were recruited into the German Infant study on the influence of Nutrition Intervention PLUS environmental and genetic influences on allergy development (GINIplus) between September 1995 and June 1998 in Munich and Wesel. Infants with at least one allergic parent and/or sibling were allocated to the interventional study arm investigating the effect of different hydrolysed formulas for allergy prevention in the first year of life.[^40^](https://paperpile.com/c/OsX7sm/Br72J) All children without a family history of allergic diseases and children whose parents did not give consent for the intervention were allocated to the non-interventional arm. Detailed descriptions of the LISAplus and GINIplus studies have been published elsewhere1,2. DNA was collected at the age 6 and 10 years and 1511 children from the Munich study center from both studies were genotyped. For both studies, approval by the local Ethics Committees and written consent from participant's families were obtained.

**Acknowledgements and Funding**

The authors thank Martina Müller-Nurasyid for her help with imputation and analysis, all families for participation in the studies and the LISAplus and GINIplus study teams for their excellent work. The GINIplus study team wishes to acknowledge the following: Institute of Epidemiology I, Helmholtz Zentrum München, German Research Center for Environmental Health, Neuherberg (Heinrich J, Brüske I, Schulz H, Flexeder C, Zeller C, Standl M, Schnappinger M, Ferland M, Thiering E, Tiesler C); Department of Pediatrics, Marien-Hospital, Wesel (Berdel D, von Berg A); Ludwig-Maximilians-University of Munich, Dr von Hauner Children’s Hospital (Koletzko S); Child and Adolescent Medicine, University Hospital rechts der Isar of the Technical University Munich (Bauer CP, Hoffmann U); IUF- Environmental Health Research Institute, Düsseldorf (Schikowski T, Link E, Klümper C). The LISAplus study team wishes to acknowledge the following: Helmholtz Zentrum München, German Research Center for Environmental Health, Institute of Epidemiology I, Munich (Heinrich J, Schnappinger M, Brüske I, Ferland M, Lohr W, Schulz H, Zeller C, Standl M); Department of Pediatrics, Municipal Hospital “St. Georg”, Leipzig (Borte M, Gnodtke E); Marien Hospital Wesel, Department of Pediatrics, Wesel (von Berg A, Berdel D, Stiers G, Maas B); Pediatric Practice, Bad Honnef (Schaaf B); Helmholtz Centre of Environmental Research – UFZ, Department of Environmental Immunology/Core Facility Studies, Leipzig (Lehmann I, Bauer M, Röder S, Schilde M, Nowak M, Herberth G , Müller J, Hain A); Technical University Munich, Department of Pediatrics, Munich (Hoffmann U, Paschke M, Marra S); Clinical Research Group Molecular Dermatology, Department of Dermatology and Allergy, Technische Universität München (TUM), Munich (Ollert M).

## IoW

**Recruitment**

The IOWBC is a single-centre study designed to represent the community population. All children born on the Isle of Wight in a defined period were eligible for inclusion. Ethics approval was obtained from the local/national ethics committees at recruitment of the birth cohort between January 1989 and February 1990, and subsequently at each assessment. The cohort was recruited through the 1509 women who gave birth to 1536 children on the IOW during the recruitment period. All 1509 mothers were recruited and consented to complete questionnaires and provide samples soon after birth. Parental consent was obtained from 1456 of the 1536 children for inclusion into a longitudinal study of asthma and allergic disease.

**Acknowledgements and Funding**

Recruitment and initial assessment for the first 4 years of age was supported by the Isle of Wight Health Authority. The 10-year follow-up of this study was funded by the National Asthma Campaign, UK (Grant No 364) and the 18-year follow-up by NIH/NHLBI R01 HL082925-01. The 21 year assessment was funded by Medical Research Council, UK (G23369). The 26 years assessment was supported by The David Hide Asthma & Allergy Research Trust.

## INMA

**Recruitment**

Population-based birth cohorts were established as part of the INMA – INfancia y Medio Ambiente [Environment and Childhood] Project in several regions of Spain following a common protocol. This analysis uses the INMA subcohort of Menorca established between 1997 and 1998 in Menorca island. This project aims to study the associations between pre- and postnatal environmental exposures and growth, health, and development from early fetal life until adolescence and has been described previously in detail.[^44^](https://paperpile.com/c/OsX7sm/ZHrAP) Pregnant women were enrolled during the 1st trimester of pregnancy at public primary health care centers or public hospitals and children were followed after birth until 18y of age. Informed consent was obtained from all participants and the study was approved by the Hospital Ethics Committee.

**Acknowledgements and Funding**

This study was funded by grants from Instituto de Salud Carlos III (97/0588, 00/0021-02, G03/176, 04/1705, 06/1756, 09/1958 and PS09/00432), Fundació La Marató de TV3, Obra Social la Caixa (IV Convocatoria de ayudas a la Investigación en Enfermedades Neurodegenerativas) and European Community (Contract No. QLK4-CT-2000-00263). DNA extractions and genotyping was performed at the Spanish National Genotyping Centre (CEGEN-Barcelona). The authors would particularly like to thank all the participants for their generous collaboration. The authors are grateful to Mireia Garcia, Maria Victoria Estraña, Maria Victoria Iturriaga, Cristina Capo and Josep LLuch for their assistance in contacting the families and administering the questionnaires. A full roster of the INMA Project Investigators can be found at <http://www.proyectoinma.org/presentacion-inma/listado-investigadores/en_listado-investigadores.html>.

## MAAS

**Recruitment**

The Manchester Asthma and Allergy Study is an unselected (i.e. population-based), birth cohort study. The setting is the maternity catchment area of Wythenshawe and Stepping Hill Hospitals, comprising of 50 square miles of South Manchester and Cheshire, UK, a stable mixed urban-rural population. Study was approved by the Local Research Ethics Committee. Informed consent was obtained from all parents.

*Screening & Recruitment*

All pregnant women were screened for eligibility at antenatal visits (8th-10th week of pregnancy). The study was explained to the parents, and informed consent for initial questionnaires and skin prick testing was obtained. Both parents completed a questionnaire about their and their partner’s history of asthma and allergic diseases and smoking habits. If the pregnant woman’s partner was not present at the antenatal clinic visit, an invitation was sent for him to attend an open-access evening clinic for skin prick testing and questionnaire. Once both parents had completed questionnaires and skin prick testing, a full explanation of the proposed future follow-up for the child was given. Of the 1499 couples who met the inclusion criteria (<10 weeks of pregnancy, maternal age >18 years, questionnaire and skin test data available for both parents), 288 declined to take part in the study. A total of 1185 participants had at least some evaluable data. *Follow-up:* The children have been followed prospectively, and attended review clinics at ages 1, 3, 5, 8 and 11 years.

**Acknowledgements and Funding**

We would like to thank the children and their parents for their continued support and enthusiasm. We greatly appreciate the commitment they have given to the project. We would also like to acknowledge the hard work and dedication of the study team (post-doctoral scientists, research fellows, nurses, physiologists, technicians and clerical staff). This report includes independent research supported by National Institute for Health Research Respiratory Clinical Research Facility at Manchester University NHS Foundation Trust (Wythenshawe). The views expressed in this publication are those of the author(s) and not necessarily those of the NHS, the National Institute for Health Research or the Department of Health. MAAS was supported by the Asthma UK Grants No 301 (1995-1998), No 362 (1998-2001), No 01/012 (2001-2004), No 04/014 (2004-2007), the BMA James Trust and Medical Research Council, UK (G0601361) and The Moulton Charitable Foundation (2004-current); the Medical Research Council (MRC) Grants G0601361, MR/K002449/1 and MR/L012693/1, and Angela Simpson is supported by the NIHR Manchester Biomedical Research Centre. The authors would like to acknowledge the North West Lung Centre Charity for supporting this project.

## ISAAC II / MAGICS

**Recruitment**
This study was performed using asthma cases derived from the Multicenter Asthma Genetics in Childhood (MAGIC) study (E1) and reference children randomly selected from the cross-sectional International Study of Asthma and Allergies in Childhood Phase II (ISAAC II) in Munich and Dresden (E2). Written informed consent was obtained from the parents of all children from both populations and the study protocols were approved by the ethics committees of each participating center.
The ISAAC II study (E2) was conducted in Germany (Munich and Dresden) to assess the prevalence of asthma in school children at 9–11 years of age. A random subset of 800 German children was selected for a GWA study as previously described (E3). This random subset was investigated in this study for BHR, atopy, allergic rhinitis and atopic eczema and served as a reference to asthma cases derived from the MAGIC study in the GABRIEL consortium GWA study.

**Acknowledgements & funding**

Supported by the German ministry of education and research (BMBF) as part of the National Genome Research Network (NGFN), with grant [NGFN 01GS0810](https://www.sciencedirect.com/science/article/abs/pii/S0091674912026760?via%3Dihub#gs2), and by the European Commission as part of the GABRIEL (a multidisciplinary study to identify the genetic and environmental causes of asthma in the European Community) contract (no. 018996) under the Integrated Program LSH-2004-1.2.5-1. M.K. is a member of the German Center for Lung Research (DZL). R.C.P. is supported by a research fellowship from the GRK1441 (Allergy) program of Deutsche Forschungsgemeinschaft (DFG) and was supported by the PhD program “Molecular Medicine,” Hannover Biomedical Research School (HBRS).

## NTR

**Recruitment**

In the Young Netherlands Twin Register (YNTR), twins are followed from birth onwards. Around every two to three years, a survey is sent out inquiring about physical and mental health. At ages 1, 2, 3, 5, 7, 10 and 12 years, the surveys are completed by the parents and/or teachers, from age 14 onward, twins and their non-twin siblings are asked to complete the surveys by themselves. At age 5 years of the twins, parents are asked a series of questions about allergies and rhinitis.[^45^](https://paperpile.com/c/OsX7sm/R7NbF) Adult and adolescent twins (ANTR) have been recruited through city councils and media, and complete a self-rating survey every few years. Surveys contain a series of questions about doctor diagnosed diseases and disorders. Subsamples of twin families have taken part in biobank and genotyping studies.

**Acknowledgements and Funding**

The Netherlands Organization for Scientific Research (NWO 480-15-001/674): Netherlands Twin Registry Repository: researching the interplay between genome and environment; Biobanking and Biomolecular Resources Research Infrastructure (BBMRI –NL, 184.021.007); Amsterdam Public Health (APH); KNAW Academy Professor Award **(**PAH/6635); the Avera Institute, Sioux Falls, South Dakota (USA) and Grand Opportunity grants 1RC2 MH089951 and 1RC2 MH089995).

## RAINE

**Recruitment**

The Western Australian Pregnancy Cohort (Raine) Study is a prospective pregnancy cohort where 2900 were recruited from King Edward Memorial Hospital between 1989 and 1991. Data were collected throughout pregnancy and the children have been followed-up at ages 1, 2, 3, 5, 8, 10, 14, 17, 18, 20, and 23. Ethics approval for this study was obtained from King Edward Memorial Hospital and Princess Margaret Hospital. Participants were consented to being involved in this study prior to each follow-up.

**Acknowledgements and Funding**

This study was supported by the National Health and Medical Research Council of Australia [grant numbers 572613, 403981 and 003209] and the Canadian Institutes of Health Research [grant number MOP-82893]. The authors are grateful to the Raine Study participants and their families, and to the Raine Study research staff for cohort coordination and data collection. The authors gratefully acknowledge the NH&MRC for their long term contribution to funding the study over the last 25 years and also the following Institutions for providing funding for Core Management of the Raine Study: The University of Western Australia (UWA), Curtin University, Raine Medical Research Foundation, UWA Faculty of Medicine, Dentistry and Health Sciences, The Telethon Kids Institute, Women and Infants Research Foundation (King Edward Memorial Hospital), Murdoch University, The University of Notre Dame (Australia), and Edith Cowan University. The authors gratefully acknowledge the assistance of the Western Australian DNA Bank (National Health and Medical Research Council of Australia National Enabling Facility).

This work was supported by resources provided by the Pawsey Supercomputing Centre with funding from the Australian Government and the Government of Western Australia

## PIAMA

**Recruitment**

PIAMA is a birth cohort study consisting of two parts: a placebo controlled intervention study in which the effect of mite impermeable mattress covers was studied and a natural history study in which no intervention took place. Details of the study design have been published previously. Recruitment took place in 1996-1997. A screening questionnaire was distributed to pregnant women visiting one of 52 prenatal clinics at three regions in the Netherlands. A total of 10,232 pregnant women completed a validated screening questionnaire. Mothers reporting a history of asthma, current hay fever or allergy to pets or house dust mite were defined as allergic. Based on this screening, 7862 women were invited to participate, of whom 4,146 women (1327 allergic and 2819 nonallergic) gave written informed consent. The response rates to the annual questionnaires ranged from 3030 (92%) at age 1 to 2732 (83%) at age 8 years. DNA was collected from 2162 children at age 4 and/or 8 years. Genome-wide genotyping was performed within the framework of the Gabriel Consortium. For this study, DNA samples from 213 children with parental reported doctor diagnosed asthma ever at age 8 years and from 213 controls without doctor diagnosed asthma or wheeze ever at age 8 years were provided. From these children, 50 cases (≥ 1 skin test positive) and 176 controls were selected. Specific allergens tested for were Dermatophagoides pteronyssinus, Dermatophagoides farinae, Alternaria alternata, mixed grass pollen, mixed tree pollen, cat, and dog. Skin test positivity was defined as one or ore positive skin test with a diameter of 3 mm of larger. For specific IgE to inhalant allergens, we included 161 cases (≥ 0.35 IU/ml) and 176 controls. We tested by Radio Allergo Sorbent Test for any of the following allergens: Dermatophagoides pteronyssinus, Fel d1, Can f1, Dactylis Glomerata, Betula verrucosa, Alternaria Alternata, milk and egg. Controls were defined as children without specific IgE (<0.35 Iu/ml) to these allergens. Genotyping was performed with an Illumina Human610 quad array. SNPs were excluded that fullfilled one or more of the following criteria: p-value for test of Hardy-Weinberg equilibrium £ 1E -7, genotyping call rate ><95% or MAF >< 1%. SNPs were imputed with IMPUTE version 2 software using HAPMAP CEU release #22 b36. Genome-wide association analyses were performed using SNPTEST version 1.1.5. The Medical Ethical Committees of the participating institutes approved the study.

**Acknowledgements & funding**

The PIAMA study would like to thank all participants, and co-investigators of the study. The PIAMA study is supported by the Dutch Asthma Foundation (grant 3.4.01.26, 3.2.06.022, 3.4.09.081 and 3.2.10.085CO), the ZonMw (a Dutch organization for health research and development; grant 912-03- 031), and the ministry of the environment. Genome-wide genotyping was funded by the European Commission as part of GABRIEL (A multidisciplinary study to identify the genetic and environmental causes of asthma in the European Community) contract number 018996 under the Integrated Program LSH-2004-1.2.5-1 Post genomic approaches to understand the molecular basis of asthma aiming at a preventive or therapeutic control.

## MOBA

**Recruitment**
Norway has ∼4.5 million inhabitants, and ∼55 000 births a year. The target population of the study is all women who give birth in Norway. There are no exclusion criteria. All hospitals and maternity units with more than 100 births annually, altogether 52 units, are to be included, and by January 2006, 50 units participate in the study. For practical reasons, the sampling frame comprises pregnant women who attend routine ultrasound examination. Together with appointments for ultrasound scanning in week 17–18 of pregnancy, the pregnant women receive a postal invitation that includes an informed consent form, the first questionnaire, an information brochure as well as consent form and questionnaire for the father.

The pregnancy is the unit of observation, and a woman can participate in the study with more than one pregnancy. Each pregnancy is given an identification number, and all other data, be it from the mother, father, or the child, are linked to this number. A participant is a pregnant woman who has sent in a written informed consent to participate. The consent states that biological material is only to be used for the study of causes of disease and that analyses can also be performed in other countries. No laboratory results will be provided to the participants. They agree that MoBa can access health registries as long as it is within the general aim of the study. The participants can choose to withdraw at any time (will not receive more questionnaires) or to be deleted from the study (so that all data are deleted). New sub-projects require a new written consent if they imply direct contact with the participants. A detailed protocol of the study including the consent can be found elsewhere (<http://www.fhi.no/morogbarn>).

**Acknowledgements & funding**

The donations of questionnaire data and biological material from MoBa participants are gratefully acknowledged. The study is supported by The Norwegian Ministry of Health, NIH/NIEHS (grant no. N01-ES–85433), NIH/NINDS (grant no. 1 UO1 NS 047537) and Norwegian Research Council/FUGE (grant no. 151918/S10).

## SWS

**Recruitment**

Participants were mother-child pairs from the Southampton Women’s Survey; a cohort study with the objective, among others, of studying early life environmental influences on child growth and development, including respiratory disease and asthma in childhood[[18](https://www.ncbi.nlm.nih.gov/pmc/articles/PMC3689612/#R18)]. During the period 1998-2002, 12,579 women aged 20-34 were recruited through their GP surgeries prior to conception. There were 1973 births before the end of 2003. Those born before 35 weeks and/or twins were excluded from the study and only one child per SWS mother was included. Parental consent was obtained and ethics approval was granted by the Southampton and South West Hampshire Local Research Ethics Committee (LREC Number 276/97, 307/97, 089/99, 125/98 and 06/Q1702/104).

**Acknowledgements & funding**

We thank the general practitioners and midwives in Southampton for their assistance in the SWS. Many staff make up the SWS Study Group and we are grateful for all their work in recruiting and interviewing the participants, and processing the data and the samples. Finally, we are extremely grateful to the participants in the SWS who have given so much time and information about themselves to the Survey. The SWS is grateful for financial support from the UK Medical Research Council, the University of Southampton, the Dunhill Medical Trust, the British Heart Foundation, and the Food Standards Agency. Additional support for ancillary studies has been received from the BUPA Foundation, Hope, Cancer Research UK, the Biotechnology and Biological Sciences Research Council, the Arthritis Research Campaign, SPARKS (Sport Aiding medical Research for KidS), the British Lung Foundation, the National Osteoporosis Society of Great Britain, the Research Council of Norway, and the US National Institutes of Health.

## SEATON

**Recruitment**
The database from the 15 year assessment will be used to contact participants by post. Non-respondents will be contacted a second time by post. We will ask staff in Data Management at the University of Aberdeen to identify and anonymously pass on letters of invitation to participants who do not respond to the second mailing. Participants will be asked to complete a respiratory questionnaire (online or by paper). We anticipate approximately 700 of the original cohort members to actively take part in this study and to get a more complete dataset, we will seek approval from the Grampian Safe Haven and Information Service Division to link the SEATON cohort to GP prescribing to identify those children who have developed asthma (identified by receipt of inhaled steroids).

**Acknowledgements & funding**

The authors thank the midwifery staff of the Aberdeen Maternity Hospital, Mr. George Henderson, Mrs. Alison Scaife, and Mrs. Gillian Moir for collecting and analyzing the cord blood samples, and all the mothers and children who have taken part in the study. This study was funded by grants from Asthma UK and Tenovus Scotland neither of whom contributed to the study design, data analysis or preparation of this manuscript. The researchers are independent of Asthma UK and Tenovus Scotland.

# **Imputation of GWAS summary statistics**

To increase the overlap between our summary results and the published GWAS we utilized Functionally-Informed Z-score Imputation (<https://pypi.org/project/pyfizi/>, <https://github.com/bogdanlab/fizi>) to impute up to 722,277 additional variants based on the 1000 genomes panel (phase 3), relying on the Gaussian Imputation Algorithm [(3)](https://www.zotero.org/google-docs/?mDEG0t). FIZI-imputed variants with an imputation accuracy (*r^2^pred*) less than 0.9 were excluded from the analysis. Apart from increasing genotype density of the summary results, FIZI imputation also converts variant estimates to Z-scores. See **supplementary table 4** for more details on the number of imputed variants. We converted Z-scores back to effect sizes using the following equations from Zhu et al. 2016 [(4)](https://www.zotero.org/google-docs/?aGbch1).

$$\hat{b}=z/\sqrt{2p(1-p)(n+z^{2}}$$

$$SE=1/\sqrt{2p(1-p)(n+z^{2})}$$

Where *p* is the minor allele frequency of the SNP, *z* is the z-score and *n* is the sample size.

# **Data Visualization**

Data analysis and visualization was conducted in R (version 4.2.2). Volcano plots, forest plots and heatmaps were created using ‘ggplot2’ [(5)](https://www.zotero.org/google-docs/?vCvGoR), while Manhattan plots were created using the R-package ‘ggfastman’.

# **Genetic Similarity Between Adult Traits and Preschool wheeze**

Genetic correlation was approximated with GSMR by fitting a slope between coefficients of lead variants from the related traits and the coefficients of the same variants for the wheeze phenotypes. The intercept was fixed at zero to ensure the slope reflected a proportional relationship between variant coefficients. Each variant is weighted with its strength of association in both traits while accounting for residual linkage disequilibrium (LD) to decrease signal inflation. Variants were included if they were significantly associated with the related traits (P < 5e-8) and not in strong LD with each other (r^2^ < 0.05). Coefficients estimated from GSMR can be estimated as odds-ratios, since the outcome GWAS is binary phenotype. No conditional analysis was performed to exclude variants in residual LD, since GSMR accounts for weak correlations between the included variants [(6)](https://www.zotero.org/google-docs/?TCRFBt) (see Supplementary Code).

# **Published GWAS on Asthma and Asthma-related Traits in Adulthood**

Shared genetic influence was characterized by utilizing existing knowledge of genetic risk factors for asthma related traits in adulthood, such as adult onset asthma, atopy (atopic dermatitis & allergic sensitization), eosinophil count and lung function.

## Asthma ever in Global Biobank Meta-analysis initiative

See supplementary data under cohort description in Tsuo et al. 2022 for complete description of asthma ever for each cohort in the meta-analysis. We only included individuals of European descent [(7)](https://www.zotero.org/google-docs/?dbXBIc).

## Childhood vs. adult onset asthma in UK BIobank

We used the age at first diagnosis reported in fields 3786 and 22147 (the age was averaged when values in both fields were available. Those diagnosed before 19 were assigned to childhood onset asthma (COA, N = 14,873) and those diagnosed between 20 & 60 years of age were assigned to adult onset asthma (AOA, N = 26,582). A set of 300,671 controls who did not suffer from any allergic disease (asthma, hay fever, eczema, or other allergies) was used as controls [(8)](https://www.zotero.org/google-docs/?AOMGuw).

## Asthma exacerbations (0-6 year)

Children with repeated acute hospitalizations for asthma (ICD8 codes 493, ICD-10 codes J45-46) between ages 2 to 6 years were identified from the Danish National Patient Register. Inclusion criteria required at least two hospitalizations lasting >1 day, with a minimum 6-month gap between episodes. Exclusions included comorbidities, chronic diagnoses affecting asthma risk, low birth weight (<2.5 kg), or gestational age <36 weeks. Cases were characterized based on asthma and acute bronchitis hospitalizations and concurrent atopy.

Control individuals (5328 non-asthmatic) were from the Inter99 cohort, a Danish study on ischemic heart disease prevention. Asthma cases in the iPSYCH study were defined using National Patient Registries data, identifying those with at least one hospitalization for asthma exacerbations in the first 6 years of life (ICD8-codes 493, ICD-10 codes J45-46). Individuals without such hospitalizations served as controls in both studies. In the combined dataset, 525,976 SNPs remained after quality control [(9,10)](https://www.zotero.org/google-docs/?RQyuOl).

## Atopic dermatitis

In FinnGen, cases needed ICD-10 code L20, ICD-9 code 6918, or ICD-8 code 691 in health registries, excluding ICD-9 code 6918X. Those lacking these codes were controls. In Estonian and UK Biobanks, L20 indicated AD cases, while no L20 record meant controls.

Due to heterogeneity in AD definitions, FinnGen conducted sensitivity analyses with stricter criteria: (1) ICD-10 code L20.0, ICD-9 code 6918B, or ICD-8 code 69100 ("AD strict"), and (2) KELA reimbursement codes 134, 395, or 317 ("AD reimb"). "AD strict" had 6,412 cases and 253,666 controls, while "AD reimb" had 6,739 cases and 236,030 controls [(11)](https://www.zotero.org/google-docs/?LQrIjr).

## Allergic sensitization

See supplementary data under cohort description in Waage et al. 2018 for complete description of allergic sensitization for each cohort in the meta-analysis [(7)](https://www.zotero.org/google-docs/?XqgfHH).

## Eosinophil count

Eosinophil count was determined by flow cytometry gates. Samples were excluded based on certain criteria: pregnancy, acute medical/surgical illness, blood cancer, leukemia, lymphoma, chemotherapy, myelodysplastic syndrome, bone marrow transplant, congenital/hereditary anemia (e.g., sickle cell anemia or thalassemia), HIV, end-stage kidney disease, dialysis, EPO treatment, splenectomy, cirrhosis, and extreme measurements (e.g., WBC count > 100∗10^9/L with > 5% immature cells or blasts). WBC subtypes were represented using relative counts, and raw phenotypes were regressed on age, age-squared, sex, principal components, and cohort-specific covariates. WBC-related traits were log10 transformed before regression, and residuals were inverse normalized for cohort-level association analysis or GWAS [(12)](https://www.zotero.org/google-docs/?Ni0a5A).

## Lung function measurements

Lung function measurements were derived from spirometry measurements in UK Biobank. We excluded FVC measurements from our analysis due to low associated with airway obstruction and thus not believed to be asthma related [(13)](https://www.zotero.org/google-docs/?G9lv8i).

# **Opposite Direction of Effects: Analysis of Known Asthma Variants**

A small group of variants previously associated with asthma were also associated with wheeze phenotypes but with opposite direction of effect, although none of them were significant after adjusting for multiple testing. One SNP with opposite direction worth mentioning is rs174535 in the fatty acid desaturase (FADS) cluster. The T-allele of rs174535 was previously associated with a higher risk of adult-onset asthma [(8)](https://www.zotero.org/google-docs/?dXm6y9) but was associated with a lower risk of persistent wheeze in our study. The T-allele of rs174535 is in high LD with the A-allele of rs1535 (r2 = 0.94), and both alleles tag the major haplotype in individuals of European ancestry, which is associated with higher plasma levels of n-3 long-chain polyunsaturated fatty acids (LCPUFA), including DHA and EPA [(14)](https://www.zotero.org/google-docs/?7fyhh8). In keeping with our results, a previous study found that children carrying the minor alleles at this locus (G allele of rs1535), and thereby lower levels of EPA and DHA, had increased risk of childhood asthma that was abrogated by higher dietary intake of EPA and DHA [(15)](https://www.zotero.org/google-docs/?ZpeE3t). Similarly, a randomized clinical trial with fish oil supplements to pregnant mothers showed an increased risk of persistent wheeze in the children of mothers carrying the LCPUFA-reducing G-allele of rs1535, which was abrogated by the pregnancy supplementation [(16)](https://www.zotero.org/google-docs/?IpNZyu). This suggests that genetic variation in the FADS region, and resultant changes in LCPUFA levels, has opposite effects on adult-onset asthma and preschool wheezing.

# **Supplementary code**

**Supplementary code 1.** Command line parameters for the GSMR tool in the analysis of pleiotropic associations of related traits to wheezing phenotypes. The commands signify that SNPs with p-values less than 5e-8 are taken forward in the analysis, but SNPs in LD with less than 0.05 are also included. The cutoff for the HEIDI test is set to zero, meaning that no variants will be filtered away. Chance correlations and mismatches in MAF between reference genome and summary stats are still considered using the –diff-freq and –gsmr-ld-fdr flags.

gcta64 \

--bfile g1000_eur \

--gsmr-file related_traits.txt wheeze_phenotypes.txt\

--gsmr-direction 0 \ # Direction: related-trait -> wheeze

--clump-r2 0.05 \ # Remove variants with r2 > 0.05

--clump-kb 1000 \ # Remove variants within a 1MB window

--gwas-thresh 5e-8 \ # Only include variants with P < 5e-8 (related trait)

--diff-freq 0.2 \ # Remove variants with large difference in MAF

--heidi-thresh 0 \ # Turn off HEIDI pleiotropy-filter (P < 0)

--gsmr-snp-min 3 \ # Only perform analysis if 10 variants pass criteria

--gsmr-ld-fdr 0.05 \ # Account for chance correlations

--out genetic_overlap \

# References

[1.	Fraser A, Macdonald-Wallis C, Tilling K, Boyd A, Golding J, Davey Smith G, et al. Cohort Profile: The Avon Longitudinal Study of Parents and Children: ALSPAC mothers cohort. Int J Epidemiol. 2013 Feb 1;42(1):97–110.](https://www.zotero.org/google-docs/?ngR3uE)

[2.	Boyd A, Golding J, Macleod J, Lawlor DA, Fraser A, Henderson J, et al. Cohort Profile: The ‘Children of the 90s’—the index offspring of the Avon Longitudinal Study of Parents and Children. Int J Epidemiol. 2013 Feb;42(1):111–27.](https://www.zotero.org/google-docs/?ngR3uE)

[3.	Pasaniuc B, Zaitlen N, Shi H, Bhatia G, Gusev A, Pickrell J, et al. Fast and accurate imputation of summary statistics enhances evidence of functional enrichment. Bioinformatics. 2014 Oct 15;30(20):2906–14.](https://www.zotero.org/google-docs/?ngR3uE)

[4.	Zhu Z, Zhang F, Hu H, Bakshi A, Robinson MR, Powell JE, et al. Integration of summary data from GWAS and eQTL studies predicts complex trait gene targets. Nat Genet. 2016 May;48(5):481–7.](https://www.zotero.org/google-docs/?ngR3uE)

[5.	Wickham H. ggplot2: Elegant Graphics for Data Analysis [Internet]. Springer-Verlag New York; 2016 [cited 2022 Feb 10]. Available from: https://ggplot2.tidyverse.org](https://www.zotero.org/google-docs/?ngR3uE)

[6.	Zhu Z, Zheng Z, Zhang F, Wu Y, Trzaskowski M, Maier R, et al. Causal associations between risk factors and common diseases inferred from GWAS summary data. Nat Commun. 2018 Jan 15;9(1):224.](https://www.zotero.org/google-docs/?ngR3uE)

[7.	Waage J, Standl M, Curtin JA, Jessen LE, Thorsen J, Tian C, et al. Genome-wide association and HLA fine-mapping studies identify risk loci and genetic pathways underlying allergic rhinitis. Nat Genet. 2018 Aug;50(8):1072.](https://www.zotero.org/google-docs/?ngR3uE)

[8.	Pividori M, Schoettler N, Nicolae DL, Ober C, Im HK. Shared and distinct genetic risk factors for childhood-onset and adult-onset asthma: genome-wide and transcriptome-wide studies. Lancet Respir Med. 2019 Jun;7(6):509–22.](https://www.zotero.org/google-docs/?ngR3uE)

[9.	Ahluwalia TS, Eliasen AU, Sevelsted A, Pedersen CET, Stokholm J, Chawes B, et al. FUT2–ABO epistasis increases the risk of early childhood asthma and Streptococcus pneumoniae respiratory illnesses. Nat Commun. 2020 Dec 16;11:6398.](https://www.zotero.org/google-docs/?ngR3uE)

[10.	Bønnelykke K, Sleiman P, Nielsen K, Kreiner-Møller E, Mercader JM, Belgrave D, et al. A genome-wide association study identifies CDHR3 as a susceptibility locus for early childhood asthma with severe exacerbations. Nat Genet. 2014 Jan;46(1):51–5.](https://www.zotero.org/google-docs/?ngR3uE)

[11.	Sliz E, Huilaja L, Pasanen A, Laisk T, Reimann E, Mägi R, et al. Uniting biobank resources reveals novel genetic pathways modulating susceptibility for atopic dermatitis. J Allergy Clin Immunol. 2022 Mar;149(3):1105-1112.e9.](https://www.zotero.org/google-docs/?ngR3uE)

[12.	Vuckovic D, Bao EL, Akbari P, Lareau CA, Mousas A, Jiang T, et al. The Polygenic and Monogenic Basis of Blood Traits and Diseases. Cell. 2020 Sep 3;182(5):1214-1231.e11.](https://www.zotero.org/google-docs/?ngR3uE)

[13.	Shrine N, Guyatt AL, Erzurumluoglu AM, Jackson VE, Hobbs BD, Melbourne CA, et al. New genetic signals for lung function highlight pathways and chronic obstructive pulmonary disease associations across multiple ancestries. Nat Genet. 2019 Mar;51(3):481–93.](https://www.zotero.org/google-docs/?ngR3uE)

[14.	Lemaitre RN, Tanaka T, Tang W, Manichaikul A, Foy M, Kabagambe EK, et al. Genetic loci associated with plasma phospholipid n-3 fatty acids: a meta-analysis of genome-wide association studies from the CHARGE Consortium. PLoS Genet. 2011 Jul;7(7):e1002193.](https://www.zotero.org/google-docs/?ngR3uE)

[15.	Talaei M, Sdona E, Calder PC, Jones LR, Emmett PM, Granell R, et al. Intake of *n* -3 polyunsaturated fatty acids in childhood, *FADS* genotype and incident asthma. Eur Respir J. 2021 Sep;58(3):2003633.](https://www.zotero.org/google-docs/?ngR3uE)

[16.	Bisgaard H, Stokholm J, Chawes BL, Vissing NH, Bjarnadóttir E, Schoos AMM, et al. Fish Oil–Derived Fatty Acids in Pregnancy and Wheeze and Asthma in Offspring. N Engl J Med. 2016 Dec 29;375(26):2530–9.](https://www.zotero.org/google-docs/?ngR3uE)
